# Evaluation of the contribution of the minor envelope complex glycoprotein 3 ectodomain to the porcine reproductive and respiratory syndrome virus 1 neutralizing antibody response

**DOI:** 10.1099/jgv.0.002328

**Published:** 2026-09-03

**Authors:** Jack W. P. Hayes, Jane C. Edwards, Kristel Ramirez Valdez, Sylvia Crossley, Rory C. F. de Brito, Danish Munir, Kevan Hanson, Kostas Paschos, Krunal Polra, Jeongho An, Jay G. Calvert, Marie Bonnet Di Placido, Jonathan F. Lovell, Robin Shattock, John A. Hammond, Raymond J. Owens, Simon P. Graham

**Affiliations:** 1 The Pirbright Institute, Woking, UK; 2 Division of Structural Biology, Nuffield Department of Medicine, University of Oxford, Oxford, UK; 3 Imperial College, London, UK; 4 Zoetis Inc., Kalamazoo, MI, USA; 5 University of Buffalo, Buffalo, NY, USA; 6 Rosalind Franklin Institute, Harwell, UK

**Keywords:** porcine reproductive and respiratory syndrome virus (PRRSV), glycoprotein 3 (GP3), porcine monoclonal antibodies, PRRSV neutralizing antibodies, vaccine design for PRRSV

## Abstract

Porcine reproductive and respiratory syndrome viruses (PRRSVs) cause significant economic losses to the global swine industry. Current live attenuated vaccines are unable to effectively control PRRSV infection, and strain variability, driven by rapid mutation, complicates the development of improved vaccines. Identification of the targets of broadly neutralizing antibodies is crucial for guiding next-generation vaccine design, yet PRRSV glycoproteins bearing conserved neutralizing epitopes remain poorly defined. To address this, recombinant soluble forms of PRRSV-1 envelope glycoproteins, GP2, GP3 and GP4, were produced using mammalian cells. The screening of serum from hyperimmune pigs demonstrated GP3 as the most highly recognized of the three glycoproteins. Fluorescently tagged GP3-tetramers were utilized to isolate single B cells from a hyperimmune pig via flow cytometry. Immunoglobulin VH and VL regions were amplified by RT-PCR and sequenced. Fifteen unique heavy and light chain pairs were cloned and expressed as recombinant monoclonal antibodies (mAbs). mAbs were evaluated for specificity, cross-reactivity and neutralizing capacity. Antigenic sites targeted by GP3-specific mAbs were mapped to several conserved linear sequences, but mAbs did not exhibit virus neutralizing activity *in vitro*, suggesting that these may serve as decoy epitopes. An immunogenicity study with recombinant GP3, delivered as a protein subunit or via an RNA vector, also demonstrated a lack of neutralizing antibody response despite high GP3-binding antibody titres. Collectively, these data suggest that antibodies directed against the isolated PRRSV-1 GP3 ectodomain are unlikely to contribute substantially to neutralization. However, the workflow established for isolating porcine mAbs could be utilized in future research to further dissect the neutralizing antibody response to PRRSV.

## Data Availability

All data supporting the findings of this study are included within the article and its supplementary material. New sequence data are publicly available: recombinant PRRSV-1 sGP3-specific monoclonal antibodies – GenBank accessions PZ206307–PZ206322, and recombinant PRRSV-1 GP3 for saRNA-vectored vaccine candidate – GenBank accession PZ206323. Supplemental material is available from the Center for Open Science Open Science Framework (OSF) repository, DOI 10.17605/OSF.IO/3WAFN. The original data can be provided upon request to the corresponding author, Simon Graham (simon.graham@pirbright.ac.uk).

**Impact Statement**

PRRSV strains are antigenically diverse, which is a major challenge for next-generation vaccine design. Neutralizing antibodies targeting conserved epitopes on PRRSV envelope glycoproteins are likely essential for effective immunity. However, the identity of glycoproteins bearing such epitopes remains poorly defined. Recombinant soluble forms of PRRSV-1 minor envelope glycoproteins were assessed for antigenicity using serum from pigs sequentially infected with diverse PRRSV strains. Since GP3 was well recognized, antigen tetramers were constructed to sort single antigen-specific B cells; however, the resulting GP3-specific recombinant monoclonal antibodies (mAbs) failed to neutralize PRRSV. Immunization of pigs with GP3 as protein or via an RNA vector failed to induce a neutralizing antibody response, despite high antigen-specific antibody titres. Thus, the isolated GP3 ectodomain did not induce neutralizing antibody responses in the systems evaluated here. However, these approaches should enable the isolation of broadly neutralizing mAbs, which could be used to define conserved epitopes on PRRSV glycoproteins.

## Introduction

Porcine reproductive and respiratory syndrome viruses (PRRSV) represent one of the greatest threats to the swine industry worldwide, causing an estimated loss of over 650 million USD per annum in the USA alone [1, 2]. Clinical features of PRRSV infection manifest twofold: reproductive failure and respiratory disease, with the former primarily impacting breeding-age gilts and sows, and the latter particularly impacting young, growing and finishing pigs [3]. PRRS reproductive failure is characterized by increased abortion rates, up to a 40% incidence rate in late pregnancy [4], and the birth of mummified and weak piglets, which often succumb to respiratory disease [5]. PRRS in growing pigs is characterized by dyspnoea, lethargy, anorexia/weight loss, reduced growth performance due to systemic disease, cyanosis of the ears and PRRS-induced interstitial pneumonia [1, 5].

PRRSV exists as two species: *Betaarterivirus europensis* (PRRSV-1) and *Betaarterivirus americense* (PRRSV-2), which share ~60% nucleotide sequence identity [6]. For both species, the PRRSV genome, ~15 kb in size, encodes at least 10 ORFs, flanked by the 5′ and 3′ UTRs. ORF1a and 1b comprise the majority of the genome [2] and encode polyproteins that are cleaved to generate the non-structural proteins required for transcription and replication [7]. ORFs 2–6 encode for the structural proteins which make up the viral envelope: the non-glycosylated matrix (M) proteins and the N-glycosylated glycoproteins (GPs) GP2, GP3, GP4 and GP5 [2, 8]. PRRSV utilizes a unique discontinuous transcription mechanism for subgenomic mRNA (sgmRNA) transcription, enabling the generation of the small, hydrophobic envelope (E) and ORF5a proteins encoded in an alternative reading frame of sgmRNA 2 and 5, respectively [9, 10]. Proteins E, ORF5a, GP2, GP3 and GP4 form a non-covalently linked heteropentameric complex referred to as the minor envelope complex [11, 12], whereas the GP5 and M proteins form a disulphide-linked heterodimer referred to as the major envelope complex [13]. Additionally, ORF7 encodes the structural nucleocapsid (N) protein, which encapsulates the viral RNA [14, 15].

The genetic and antigenic heterogeneity of PRRSV makes control and/or eradication of PRRSV highly challenging. Neutralizing antibodies have been shown to protect against PRRSV infection, with passive transfer experiments demonstrating immunity in naïve pigs using serum from PRRSV-immune animals [16, 17]. This protection, however, was shown to be dose-dependent: while neutralizing antibody titres of 1 : 8 were able to prevent viraemia, they were insufficient to prevent tissue seeding and viral transmission, even at titres of 1 : 32, sterilizing immunity was only observed in 50% of animals tested [16]. However, PRRSV infection is characterized by a delayed and low-titre neutralizing antibody response, which likely enables viral persistence [2]. The high degree of variability among PRRSV isolates, enabled by rapid mutation and evolution, means that neutralizing antibodies that do arise are often restricted in their breadth, poorly recognizing heterologous strains [18]. Further, escape mutants can arise, evading existing neutralizing antibody responses [19, 20]. Broadly neutralizing antibodies (bnAbs) that can confer cross-protection against PRRSV strains/species are consequently an area of great interest. The development of bnAbs has been documented in pigs that had been repeatedly vaccinated or infected with PRRSV. Sera from U.S. sow herds which had been vaccinated and exposed to field strains on multiple occasions were shown to neutralize a genetically diverse range of PRRSV strains, including PRRSV-1 and -2, despite no prior evidence of PRRSV-1 exposure [19]. It has been further demonstrated that bnAb responses can be promoted through the experimental repeat inoculation of pigs with homologous PRRSV strains [21].

Vaccination strategies that can induce bnAbs and broadly protect against PRRSV infection are limited. Both modified live virus (MLV) and inactivated PRRSV vaccines are commercially available. MLV vaccines are generally preferred and have demonstrated efficacy in reducing disease severity, duration of viraemia and viral shedding [22–24]. Despite conferring protection against clinical diseases caused by closely related strains, MLVs have been shown to offer partial, or no, protection against more genetically distant strains [25]. There are also safety concerns regarding reversion of MLVs to virulence and recombination with field strains [23, 26, 27]. Inactivated PRRSV vaccines have been shown to boost immunity in sows and are therefore a useful alternative when aimed at increasing levels of maternally derived antibody [28]. Strategies to develop safer and more broadly protective PRRSV vaccines have been explored. Virus-vectored, subunit and DNA vaccines have all been evaluated as potential alternatives, typically utilizing PRRSV GPs as target antigens. In almost all instances, however, vaccine candidates developed using these techniques have proven to be equally or less effective than MLVs [25, 29–31]. Consequently, vaccines based on these techniques, though promising, are still far from practical application. Another avenue towards developing a broadly protective PRRSV vaccine is utilizing a structural vaccinology approach [32]. Elucidating neutralizing antibody epitopes is fundamental to a structure-based vaccine design. Identification of these sites is most often achieved through the isolation of monoclonal antibodies (mAbs) with broad neutralization capacity. Once epitopes have been identified, engineered *de novo* mimics can be used as immunogens to elicit antibodies with the original specificity [33]. The ability to selectively induce bnAbs against normally subdominant, conserved epitopes could overcome long-standing obstacles to the development of broadly protective PRRSV vaccines.

Neutralizing epitopes have been described in PRRSV structural proteins GP3, GP4, GP5 and M; however, there are inconsistencies between these findings, and more comprehensive studies are required to understand their contribution to a protective immune response [34, 35]. Moreover, information on PRRSV bnAbs and their cognate epitopes is scarce. Despite the promise they hold as targets for novel vaccine design [36–38], the minor envelope GPs (GP2, GP3 and GP4) remain some of the least studied PRRSV proteins. Here, we systematically evaluate the antigenicity and neutralizing relevance of recombinant soluble forms of the putative ectodomains of PRRSV-1 (s)GP2, sGP3 and sGP4, produced using mammalian cells. Based on the uniform recognition of sGP3 by antibodies from hyperimmune pigs, sGP3 tetramers were utilized to isolate mAbs, which were broadly reactive but non-neutralizing. An immunogenicity study further demonstrated a lack of a neutralizing antibody response, suggesting that the PRRSV-1 GP3 ectodomain alone does not induce a neutralizing response and may instead function as a ‘decoy’ antigen directing the B cell response away from conserved neutralizing epitopes.

## Methods

### Cells

All cell lines were cultured at 37 °C, 95% humidity and 5% CO_2_. Human embryonic kidney (HEK) 293 T cells and MARC-145 cells [the latter kindly provided by Dr Jean-Pierre Frossard, Animal and Plant Health Agency (APHA)] were grown as adherent monolayers using Dulbecco’s Modified Eagle Medium (high glucose, GlutaMAX^™^ Supplement, HEPES) (Thermo Fisher Scientific) supplemented with 10% heat-inactivated FBS (Thermo Fisher Scientific) and 1% Penicillin-Streptomycin (100 U ml^−1^ penicillin and 100 µg ml^−1^ streptomycin) (Merck Millipore). Expi293F^™^ cells (Thermo Fisher Scientific) were grown in suspension at 3–5×10^5^ cells ml^−1^ in either 125 ml or 1 l Erlenmeyer non-baffled cell culture shaker flasks (Corning^®^) on an orbital shaker at 120 r.p.m., using Expi293^™^ Expression Medium (Thermo Fisher Scientific). Porcine alveolar macrophages, obtained by post-mortem bronchoalveolar lavage of a 5-week-old PRRSV-naïve piglet, were cultured in RPMI-1640 medium (Thermo Fisher Scientific) supplemented with 10% FBS and 1% Penicillin-Streptomycin (cRPMI).

### Cloning and expression of recombinant PRRSV-1 GPs

PRRSV-1 Olot/91 ORF2 (GenBank: PZ206324), ORF3 (GenBank: PZ206325) and ORF4 (GenBank: PZ206326) sequences were codon-optimized for expression in human cells and synthesized by Integrated DNA Technologies (IDT Inc.) (Table S1, available in the online Supplementary Material). Primers were designed to generate ectodomain-only products of each PRRSV-1 minor envelope GP, without the native signal peptide (Table S2). All primers were designed with overhangs to enable cloning into the pOPIN expression vectors [39, 40].

Sequences encoding GP ectodomain regions were amplified by PCR using Q5^®^ High-Fidelity DNA Polymerase (New England Biolabs) with 100 nM of the appropriate forward and reverse primers, and 20 ng of codon-optimized gene blocks as templates. The PCR step was composed of 30 s of activation at 98 °C, 30 cycles at 62 °C (98 °C for 20 s, 62 °C for 30 s and 72 °C for 30 s) and a final extension step of 2 min at 72 °C. Products were visualized via 1% agarose gel and purified using the GFX PCR DNA and Gel Band Purification Kit (Cytiva Life Sciences). Cloning of purified products was performed using InFusion^®^ following the ClonExpress Ultra One Step Cloning Kit (Vazyme) protocol, and the resultant plasmids were amplified in *Escherichia coli* (Stellar Supercompetent^™^ Cells, Takara Bio). DNA was extracted using an EndoFree^®^ Plasmid Maxi Kit (Qiagen), according to the manufacturer’s protocol and eluted in DNase/RNase/protease-free deionized water.

### Expression of recombinant PRRSV-1 GPs

HEK293T cells were transfected using TransIT^®^-LT1 Transfection Reagent (Mirus Bio LLC) following the manufacturer’s protocol. After 2–3 days, cell culture supernatants and cell pellets were collected for SDS-PAGE and western blot analysis. Expi293F cells were transfected across a 5-day period using PEI MAX^®^ Transfection Grade Linear Polyethyleneimine Hydrochloride (Polysciences Inc.). For *in vivo* biotinylation, cells were co-transfected with an equivalent concentration of plasmid expressing BirA ligase [41]. Culture supernatant containing the secreted sGP was harvested at 3 days post-transfection and clarified by centrifugation (5,000 ***g***) before being dialysed and concentrated via 10 kDa Polyethersulfone Tangential Flow Filtration Sartocon^®^ Cassettes (Sartorius AG). Samples were then loaded onto a 5 ml HisTrap^™^ High Performance column (Cytiva Life Sciences). The column was washed with 20 mM imidazole in PBS, and protein was eluted using 300 mM imidazole in PBS. Column elution fractions were then analysed by SDS-PAGE and Western blot, and protein-containing fractions were pooled and re-dialysed with PBS and PMSF Protease Inhibitor (Thermo Fisher Scientific). The protein concentration of the final dialysed material was determined using the Pierce^™^ BCA Protein Assay Kit (Thermo Fisher Scientific).

### Mass photometry

Briefly, the Refeyn MPOne mass photometer was calibrated using BSA, *β*-amylase and thyroglobulin standards. The calibration curve showed an R2 of 0.994 and a maximum calibration error of 20.58%. GP3 was diluted to 20 nM in 0.22 µM filtered PBS and measured in triplicate. Mass measurements were obtained by fitting the recorded contrast values to the calibration curve generated from the protein standards.

### SDS-PAGE and western blotting

Protein samples were diluted in Laemmli sample buffer (Bio-Rad Laboratories) supplemented with either 2% *β*-mercaptoethanol (Bio-Rad Laboratories) or 50 mM dithiothreitol (Thermo Fisher Scientific) and run (40A) on 12.5% polyacrylamide gels cast using the Bio-Rad Mini-PROTEAN Tetra Cell system. Gels were then evaluated either by Coomassie brilliant blue protein stain (Abcam) or western blotting. For western blot, a semi-dry transfer protocol was used, utilizing the Trans-Blot^®^ Turbo^™^ Transfer System (Bio-Rad Laboratories). PVDF membranes were blocked for 1 h at room temperature (RT) in 15 ml PBS supplemented with 5% (wt/v) skimmed milk powder and 0.1% (v/v) Tween-20 (Merck Millipore) and probed with a histidine (His) tag antibody (Bio-Rad Laboratories), followed by either polyclonal goat anti-mouse IgG-HRP (Thermo Fisher Scientific) or streptavidin-HRP (Thermo Fisher Scientific). Washes were performed using PBS supplemented with 0.1% Tween-20. Blots were developed with 3 ml Pierce^™^ ECL Plus Western Blotting Substrate (Thermo Fisher Scientific) and imaged first by bright field to capture the protein ladder, and then chemiluminescence was imaged, both using a Bio-Rad GelDoc Go Gel Imaging System. Exposure time varied for each antibody, ranging from seconds up to 10 min. Images were merged later for analysis.

### Flow cytometric cell sorting

Biotinylated recombinant sGP3 was tetramerized using Brilliant Violet 421 (BV421)-labelled streptavidin (BioLegend) or Brilliant Violet 650-labelled streptavidin (BV650; BioLegend) at a 4 : 1 molar ratio on ice as described previously [42]. A ‘decoy’ tetramer was constructed using a non-PRRSV control antigen: biotinylated SARS-CoV-2 spike protein receptor-binding domain (S-RBD) [42], kindly provided by Dr Joe Newman (The Pirbright Institute) and PerCP-Cy5.5-labelled streptavidin (BioLegend). A panel of antibodies to identify IgM-, IgG- and IgA-expressing porcine B cells was used as previously described [42], in conjunction with the tetramerized sGP3 to identify and isolate PRRSV sGP3-binding B cells from previously cryopreserved peripheral blood mononuclear cells (PBMCs). Single-colour controls for each tetramer were included by staining 1×10^6^ PBMCs with mouse anti-porcine CD8α-biotin (Cambridge Bioscience), followed by either of the three streptavidin-fluorochrome conjugates used for tetramer assembly. Tetramers were diluted 1 : 20 (v/v) before staining cells on ice, in conjunction with the antibody panel detailed above. Gates to distinguish positive tetramer events from background staining were set by staining PBMCs from a PRRSV-naïve pig. Sorted cells were collected in cell collection buffer: 0.01M Tris (pH 8.0), 100 units RNasin^®^ Ribonuclease Inhibitor (Promega), diluted in RNase-free water and frozen at −80 °C for subsequent analysis. Flow cytometric cell sorting was performed using a BD FACSAria^™^ III Cell Sorter (BD Pharmingen).

### Generation of recombinant mAbs

Reverse single-cell transcription of RNA and nested PCR of porcine IgG, Igκ and Igλ genes were adapted from a previously described protocol [43], with new primers being designed for porcine variable and constant regions of the IgG heavy-chain locus, lambda (LCλ) or kappa (LCκ) light-chain loci (Table S3). In brief, cDNA was generated from single-sorted B cells using the Sensiscript^®^ Reverse Transcription Kit (Qiagen). Each sample was taken through a reference gene PCR, testing for glyceraldehyde-3-phosphate dehydrogenase (GAPDH) amplification using primers GAPDH_F: ACCCAGAAGACTGTGGATGG and GAPDH_R: ACGCCTGCTTCACCACCTTC, and, if successfully amplified, template cDNA was taken through two rounds of nested PCRs to amplify antibody chains. PCR 1 used the following protocol: 98 °C for 30 s, 25 cycles of 98 °C for 10 s, 64 °C for 20 s and 72 °C for 20 s, followed by a final elongation step at 72 °C for 2 min. PCR products from each single cell were then run and imaged on a 1% agarose gel. Cells exhibiting amplicons for both heavy and light chains were taken forward for Sanger sequencing.

Following sequencing of VH and VL amplicons, the international ImMunoGeneTics information system (IMGT^®^) [44–46] was used to annotate each sequence, identifying variable regions, complementarity-determining regions (CDRs) and determining which germline gene segment and allele the sequences were closest in identity to. The recombinant antibodies were generated as described previously [47]. Briefly, VH and VL synthetic gene fragments (Twist Bioscience) carrying 15 bp overhangs matching the leader sequence at 5′ and corresponding constant regions at 3′ were directionally cloned into corresponding pNeoSec_SsVL_kappa, pNeoSec_SsVL_lambda and pNeoSec_SsFc_IgG1 vectors; paired VH- and VL-expressing constructs were co-transfected into Expi293F cells.

### Recombinant PRRSV GP ELISA

Assessment of antibody reactivity to recombinant PRRSV-1 sGPs was performed by adapting a previously described ELISA protocol [48]. In brief, Nunc MaxiSorp ELISA plates (Thermo Fisher Scientific) were coated with 100 ng/well of purified recombinant PRRSV-1 GP or recombinant S-RBD, diluted in 100 µl/well of carbonate–bicarbonate coating buffer, 0.6 M, pH 9.6 (Merck Millipore). Plates were blocked with PBS supplemented with 5% (wt/v) skimmed milk powder (Marvel) for 2 h at 37 °C. Porcine serum samples/mAbs, serially diluted 2-fold starting at 1 : 100 or 1 : 10, respectively, were added for 1 h at 37 °C, followed by the addition of 1 : 10,000 (v/v) goat anti-porcine IgG-HRP polyclonal antibody (Abcam) for 1 h at 37 °C. TMB Liquid Substrate System for ELISA (Merck Millipore) was added at RT for 5–10 min until colour change was observed, after which reactions were stopped with 1 M sulphuric acid solution (Thermo Fisher Scientific). Absorbances at 450 and 595 nm were read via GloMax^®^ Discover Microplate Reader (Promega). All final ELISA results presented have had the OD values from S-RBD-coated wells, as well as values at the irrelevant 595 nm wavelength, subtracted to account for background and non-specific binding. Antibody binding titres were calculated as described [49].

### Peptide ELISA

Overlapping biotinylated peptides, 16-mers offset by four residues, representing the ectodomain sequence of PRRSV-1 Olot/91 GP3 were synthesized (MIMOTOPES Pty Ltd, Australia). MaxiSorp 96-well plates were coated with 500 ng/well of NeutrAvidin Protein (Thermo Fisher Scientific) diluted in carbonate–bicarbonate coating buffer, 0.6 M, pH 9.6 (Merck Millipore), overnight at 4 °C. Plates were blocked with 200 µl/well of PBS supplemented with 5% (wt/v) skimmed milk powder for 1.5 h at 37 °C, followed by addition of peptides at 2.5 µg ml^−1^ in PBS supplemented with 1% (v/v) BSA (Merck Millipore) for 2 h at RT on a rocking table. Addition of porcine sera/mAbs, followed by HRP-conjugated secondary antibody and TMB Liquid Substrate, was performed as described above, and absorbance was read at 450 nm, as well as values at the irrelevant 595 nm wavelength, subtracted to account for background and non-specific binding.

### Virus neutralization test

Neutralization of PRRSV strains was performed by adapting a previously described virus neutralization test (VNT) [50]. Briefly, either heat-inactivated serum, serially diluted 3-fold from 1 : 2, or recombinant mAbs, serially diluted 2-fold from 10 µg ml^−1^, was incubated with 400 or 40 TCID_50_ of PRRSV-1 Olot/91 (kindly provided by Dr Jean-Pierre Frossard, APHA) or PRRSV-2 VR-2332 (ATCC) for 1 h at 37 °C. MARC-145 cells or porcine alveolar macrophages were added to the serum/mAb-virus mixtures for 48 h at 37 °C and 5% CO_2_, and the cells were fixed and permeabilized (2% paraformaldehyde, 0.1% Triton X-100 in PBS) for 10 min at RT and blocked with 10% goat serum in PBS. The cells were stained with an anti-PRRSV N mAb (SDOW17-A, Rural Technologies Inc.). MARC-145 cells were then stained with a goat anti-mouse IgG secondary antibody conjugated to Alexa Fluor 488 (Thermo Fisher Scientific) and visualized using the Incucyte^®^ Live-Cell Analysis System (Sartorius), whereas porcine alveolar macrophages were stained with a goat anti-mouse IgG secondary antibody conjugated to HRPx (Thermo Fisher Scientific) and visualized using 3-amino-9-ethylcarbazole (AEC) (Merck). For MARC-145 cells, neutralization was calculated as a percentage by comparing the proportion of fluorescent cells to the total monolayer count using the Incucyte^®^ Live-Cell Analysis System (antibody titres were calculated as the reciprocal serum dilution that neutralized infection by 50%). For porcine alveolar macrophages, nAb titres were calculated as log_10_ of the reciprocal serum dilution that fully neutralized viral replication in 50% of the wells (ND_50_), based on AEC staining.

### Immunofluorescence staining

1.5×10^4^ cells/100 µl MARC-145 cells were seeded into a 96-well flat-bottom microplate (Thermo Fisher Scientific) and cultured for 18–24 h. Cells were then infected with 400 TCID_50_/well of PRRSV-1 Olot/91 or PRRSV-2 VR-2332 and incubated for 48 h. An additional 10-fold dilution series from 400 to 0.004 TCID_50_/well of PRRSV was also included in each assay to enable back titration. Following incubation, washes, blocking and fixation (as described above), 50 µl/well of recombinant mAbs, ranging from 10,000 to 20 ng ml^−1^, were added for 1 h at RT, followed by goat anti-porcine IgG(H+L)-Alexa Fluor 488 (Cambridge Biosciences). After final washes, fluorescent readout was measured using the Incucyte^®^ SX5 Live-Cell Analysis System (Sartorius AG).

### Depletion of recombinant PRRSV-1 sGP-binding antibodies

Serum depletions were performed to assess the contribution of GP3-specific antibodies to neutralization. For each experiment, immune and naïve sera were depleted with sGP3, S-RBD [42] to confirm assay specificity, or beads only to serve as a control. Dynabeads^™^ His-tag isolation and pulldown magnetic beads (Thermo Fisher Scientific) were prepared in accordance with the manufacturer’s protocol. Ten micrograms of each recombinant protein, or an equal volume of PBS for the beads only, were mixed in a total volume of 700 µl of binding buffer: dH_2_O supplemented with 100 mM (wt/v) sodium phosphate pH 8.0 (Thermo Fisher Scientific), 600 mM (wt/v) sodium chloride (Merck Millipore) and 0.02% (v/v) Tween-20 (Merck Millipore). Two milligrams of magnetic beads were added and incubated at RT for 10 min. Following binding, the tubes were placed in a 12-tube magnetic separation rack (New England Biolabs) for 5 min, and supernatant was discarded by pipetting. Beads were washed with PBS supplemented with 0.05% BSA, then resuspended at 1 mg ml^−1^ in the same buffer. Beads were incubated at a 3 : 1 v/v ratio with serum and incubated for 12–16 h at 4 °C on a benchtop roller, followed by another magnetic separation step. The protein-specific antibody-depleted sera were taken forward for analysis by ELISA and VNT.

### Construction and formulation of a self-amplifying RNA vector expressing PRRSV-1 GP3

A Venezuelan equine encephalitis virus plasmid DNA vector was cloned with a linear DNA fragment encoding PRRSV-1 Olot/91 GP3 ectodomain fused to the Ebola virus GP transmembrane domain (GenBank: PZ206323) (Fig. S1B) using the SapI golden-gate cloning method (New England Biolabs), and all successful clones were then confirmed by Sanger sequencing. The plasmids were then linearized with MluI-HF (New England Biolabs) at 37 °C for 3 h, followed by purification using the QIAquick PCR Purification Kit (Qiagen). The linearized plasmids were then used as templates to synthesize cleancap saRNA using the NEB Hiscribe RNA Kit (New England Biolabs) with cleancap reagent AU (Trilink BioTechnologies) at 37 °C for 2 h according to the manufacturer’s instructions. After IVT, the products were purified by LiCl precipitation. In brief, LiCl was added to the transcription reaction in equal volumes and incubated overnight at −20 °C, then centrifuged at 14,000 r.p.m. The visible RNA pellet was washed with 70% EtOH, followed by centrifugation to remove the ethanol. UltraPure H_2_O (Ambion) was added to resuspend the pellet. The concentration of saRNA was measured using a Nanodrop and stored at −80 °C until use.

saRNA was formulated with lipid nanoparticles (LNPs) by using the NanoAssemblr^™^ Ignite^™^ nanoparticle formulation system (Cytiva Life Sciences) with the RNA in the aqueous phase (pH 5.5, 50 mM sodium acetate and 100 mM sodium chloride buffer) and lipid mix in the ethanol phase using a flow rate of 8 ml min^−1^ and a 3 : 1 (RNA:lipid) flow rate ratio. A molar percentage of 35 : 16 : 46.5 : 2.5 lipid mixture was used for C12-200 (Corden Pharma), 1,2-distearoyl-sn-glycero-3-phosphocholine (Avanti^®^ Polar Lipids), cholesterol (plant-derived) (Avanti^®^ Polar Lipids) and 1,2-dimyristoyl-sn-glycero-3-phosphoethanolamine-N-[methoxy(polyethylene glycol)−2000] (ammonium salt) (DMPE-PEG2000) (Avanti^®^ Polar Lipids). LNPs were diluted 5-fold with 20 mM tris+10% sucrose and purified using sterilized centrifugal filters with 10 kDa molecular weight cut-off (Merck Millipore) at 17 °C and 2,000 ***g***. RNA concentration and encapsulation efficiency were measured using the RiboGreen assay. Size, polydispersity index and zeta potential of the LNPs were measured using the Zetasizer Nano (Malvern Instruments). The formulated saRNA was stored at −80 °C until use.

### Immunogenicity study

Nine female, Large White×Landrace×Hampshire cross-bred, 6–8-week-old, PRRSV-naïve (confirmed by ELISA and RT-qPCR screening of serum samples collected on the high health status supplier farm) piglets were randomly assigned by weight (RANDOM.ORG - List Randomizer) to three treatment groups (*n*=3). As this was a pilot study, a formal power calculation was not needed. However, a group size of three pigs should identify vaccine candidates unlikely to generate protective immune responses and allow estimation of the variability in response amongst pigs to inform the design of future experiments. Furthermore, the experimental design can be replicated as necessary, and the combined results analysed as in a block design. No criteria were used for inclusion and exclusion of animals during the experiment. After 7 days acclimatization, groups were immunized on days 0 and 28 by injection into the brachiocephalic muscle (i.m.) with 1 ml containing the following: (i) 100 µg sGP3 formulated in a water–oil–water emulsion with Montanide^™^ ISA 201 VG (ISA 201) adjuvant (Seppic); (ii) 100 µg PRRSV-1 GP3 coated on cobalt porphyrin-phospholipid (CoPoP)/3D(6-acyl)-PHAD^®^/QS-21 (CPQ) liposomes, made as previously described [51]; or (iii) 3 µg saRNA expressing membrane-anchored PRRSV-1 sGP3 formulated in LNPs. Responses were compared pre- and post-vaccination to remove the requirement for a placebo-immunized control group. Groups were co-housed to avoid potential ‘pen’ effects, and laboratory staff were blinded to the identity of the groups. Prior to vaccine formulation, purified recombinant PRRSV-1 sGP3 was assessed using the Pierce^™^ Chromogenic Endotoxin Quant Kit (Thermo Fisher Scientific) to ensure safe use for vaccination. Recombinant PRRSV-1 sGP3 was diluted to 0.2 mg ml^−1^ in sterile PBS and mixed 45/55% (v/v) with ISA 201 at 32 °C. Emulsification was performed by sheer mixing using an Ultra Turrax^®^ Tube Drive mixer (IKA-Werke) for 3 min at 1,100 r.p.m. Following mixing, the formulation was stored at 4 °C for a minimum of 1 h. The liposome formulation was prepared by mixing 2 ml of CPQ liposomes with 2 ml of 0.2 mg ml^−1^ PRRSV-1 sGP3 in PBS and incubating for 3 h at RT. Prior to vaccination, incorporation of sGP3 was assessed by Ni-NTA competition assay using His Pur Ni-NTA Magnetic Beads (Thermo Fisher Scientific). Ni-NTA beads were added to the coated liposomes, incubated for 30 min at RT and then removed by magnetic separator (Thermo Fisher Scientific). Samples were assessed by native-PAGE followed by Coomassie staining to confirm sGP3 attachment (Fig. S2). LNPs carrying the saRNA vector were first added to HEK293T cells, and surface expression was confirmed by staining with GP3 mAb (clone Hy P10-c40-c1, European Virus Archive Global, ref CCLV-RIE 1167) and flow cytometry (Fig. S3). To characterize immune responses, blood samples were collected by venipuncture of the external jugular vein into SST^™^ BD Vacutainer^®^ Tubes every 7 days for isolation of serum and analysis of antibody responses and into BD Vacutainer^®^ Heparin Tubes every 14 days for PBMC isolation and analysis of T cell responses. Data obtained from all animals and samples were included in the analysis. After 56 days, animals were euthanized; animals were first sedated by i.m. injection with a cocktail of Domitor (Medetomidine; 1 mg ml^−1^) and Zoletil (Tiletamine and Zolazepam; 50 mg ml^−1^) at a concentration of 0.5 ml/10 kg body weight, before an overdose of pentobarbital sodium anaesthetic (Pentoject; 20 ml/animal) by injection in the marginal ear vein (i.v.), followed by exsanguination. All procedures were conducted in accordance with the UK Animals (Scientific Procedures) Act 1986 under Project License PP0250508. Humane endpoints were in place and were not reached.

### IFN gamma ELISpot assay

Assessment of IFN gamma (IFN-γ) responses was performed by ELISpot assay, adapting methods that were previously described [50]. Briefly, PBMCs were plated at 2.5×10^5^ cells/well in 96-well PVDF membrane plates (Merck Millipore) coated with anti-porcine IFN-γ mAb (clone P2G10; BD Pharmingen). Cells were either left unstimulated (cRPMI), stimulated with a GP3 peptide pool at 1 µg ml^−1^ or stimulated with 5 µg ml^−1^ concanavalin A as a positive control. All experiments were performed in triplicate. Plates were incubated overnight at 37 °C and 5% CO_2_. The cells were removed, and secondary biotinylated anti-porcine IFN-γ mAb (clone P2C11, BD Pharmingen) was added. Plates were then developed with streptavidin–alkaline phosphatase and a BCIP/NBT colourimetric substrate (both Mabtech). Spots were counted using an ImmunoSpot Reader (Cellular Technology Limited), and the results were expressed as the number of IFN-γ-producing cells per 10^6^ cells minus the average number of IFN-γ-producing cells in unstimulated wells.

### Data and statistical analysis

Nucleotide sequences obtained were analysed using a standard nucleotide Basic Local Alignment Search Tool (blast). To analyse protein sequences, sequences were first translated in three reading frames using the ExPASy translate tool software; the correct in-frame sequence was then analysed using a standard protein blast. Sequence alignments, analysis and pairing of VH-VL chains were performed using the international ImMunoGeneTics information system (IMGT^®^) and mega11: Molecular Evolutionary Genetics Analysis version 11.0.9 [52]. Data were analysed using the GraphPad Prism 9.3.1 software. To compare ELISA and VNT results among different groups at the same time, a Mann–Whitney test (to compare two groups) or a Kruskal–Wallis test followed by Dunn’s multiple comparison tests (to compare more than two groups) was used. A two-way ANOVA with Geisser–Greenhouse correction followed by Tukey’s test was performed for other datasets. All flow cytometry data were analysed using FlowJo^™^ versions 6–10.8.1 Software (BD Pharmingen) (FlowJo^™^ Software for Windows, Version 10.8.1. Becton, Dickinson and Company).

## Results

### Expression of recombinant soluble PRRSV-1 minor envelope complex GPs

**Fig. 1.**
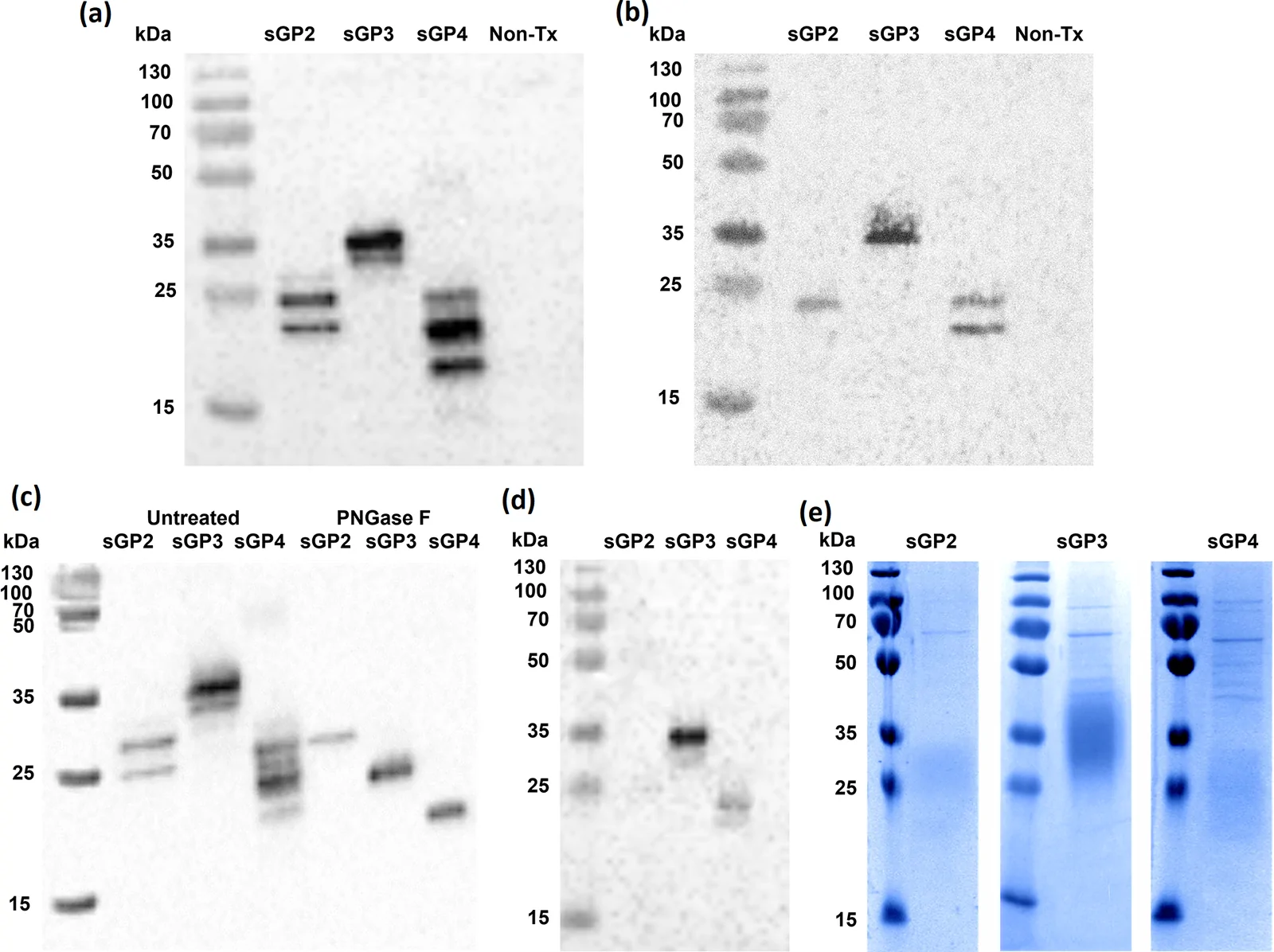
Transiently transfected HEK293T/Expi293F cell lysate and culture supernatants were assessed for expression of recombinant PRRSV-1 sGP2, sGP3 or sGP4 by western blot. Whole cell lysates (**a**) and culture supernatants (**b**) were collected 3 days post-transfection, and proteins were detected using an anti-His-HRP antibody. Protein extracted from cell lysate was treated with and without PNGase F before assessment by western blot to investigate potential N-glycosylation heterogeneity (**c**). Purification of recombinant PRRSV-1 sGPs from cell culture supernatant was performed using affinity chromatography. Eluted samples were run on an SDS-PAGE gel and then evaluated by western blot (**d**) and Coomassie protein stain (**e**) to assess purity. Non-transfected cells (non-Tx) were included as a negative control. All gels were run under reducing and denaturing conditions, using 2% *β*-mercaptoethanol and 50 mM dithiothreitol.

Small-scale cultures of HEK293T cells were first transiently transfected with the recombinant pOPIN plasmids to assess expression of PRRSV-1 sGP2, sGP3 and sGP4. Following 3 days of culture, cell lysates (Fig. 1a) and cell culture supernatants (Fig. 1b) were analysed by western blotting probing with an anti-His tag antibody, since all proteins were C-terminally His-tagged. Western blotting revealed several protein bands for each GP with molecular weights of ~25, 35 and 20 kDa for sGP2, sGP3 and sGP4, respectively, aligning with the expected molecular masses as described previously [37, 53, 54] and indicating successful expression and secretion of GP into the supernatant. Notably, stronger banding associated with sGP3 was observed in the culture supernatant, indicating higher expression compared to sGP2 and sGP4. We also observed that each sGP was consistently characterized by multiple bands, likely representing distinct levels of N-glycosylation occupancy. Analysis of the recombinant sGP sequences using the NetNGlyc 1.0 server [55] predicted two, four and four high-confidence N-linked glycosylation sites for sGP2, sGP3 and sGP4, respectively, supporting the extensive glycosylation observed experimentally. Enzymatic digestion of sGP2, sGP3 and sGP4 with PNGase F resulted in a downshift of each protein, consistent with the removal of multiple N-linked glycans predicted from the amino acid sequences, with a single band appearing at ~27, ~25 and ~20 kDa, respectively (Fig. 1c), further lending support to the hypothesis that these were different glycoforms of each GP. However, while the shifts were typically within the expected ranges suggesting successful deglycosylation, it is possible that only partial deglycosylation was achieved, leading to the generation of heterogeneous protein isoforms.

Following the successful expression of sGP2, sGP3 and sGP4 in HEK293T cells, subsequent scale-up expression in suspension Expi293F cells was performed. The eluted fractions were then pooled, concentrated and dialysed and subsequently analysed by SDS-PAGE and either western blot, probing with an anti-His tag antibody (Fig. 1d) or streptavidin-HRP (Fig. S4), or stained with Coomassie protein stain (Fig. 1e). The results demonstrate that sGP2, sGP3 and sGP4 proteins were all successfully biotinylated and were purified from cell culture supernatant with an acceptable purity. Across six batches, the greatest yields were observed for sGP3, with an average of 884.20 µg l^−1^. Lower yields were consistently observed for sGP2 and sGP4 with an average of 115.61 and 133.33 µg l^−1^, respectively. The lower intensity of the sGP2 and sGP4 bands in Coomassie-stained SDS-PAGE gels reflects their substantially lower expression yields compared with sGP3. Additionally, mass photometry was performed to assess molecular mass, homogeneity and oligomeric state of the recombinant sGP3 (Fig. S5). The results (across three independent measurements) gave an average mass of 85 kDa, suggesting that, assuming a monomeric molecular weight of around 28 kDa (plus N-linked glycans), the protein behaves as a dimer in solution. No major higher-order species were detected, suggesting that the protein appears to be relatively homogeneous, although a minor leading edge consistent with aggregation may be present. The heterogeneous occupation of the predicted N-linked glycosylation sites likely contributes to the observed mass distribution and complicates precise determination of the oligomeric state.

### Antigenicity of recombinant soluble PRRSV-1 minor envelope complex GPs

**Fig. 2.**
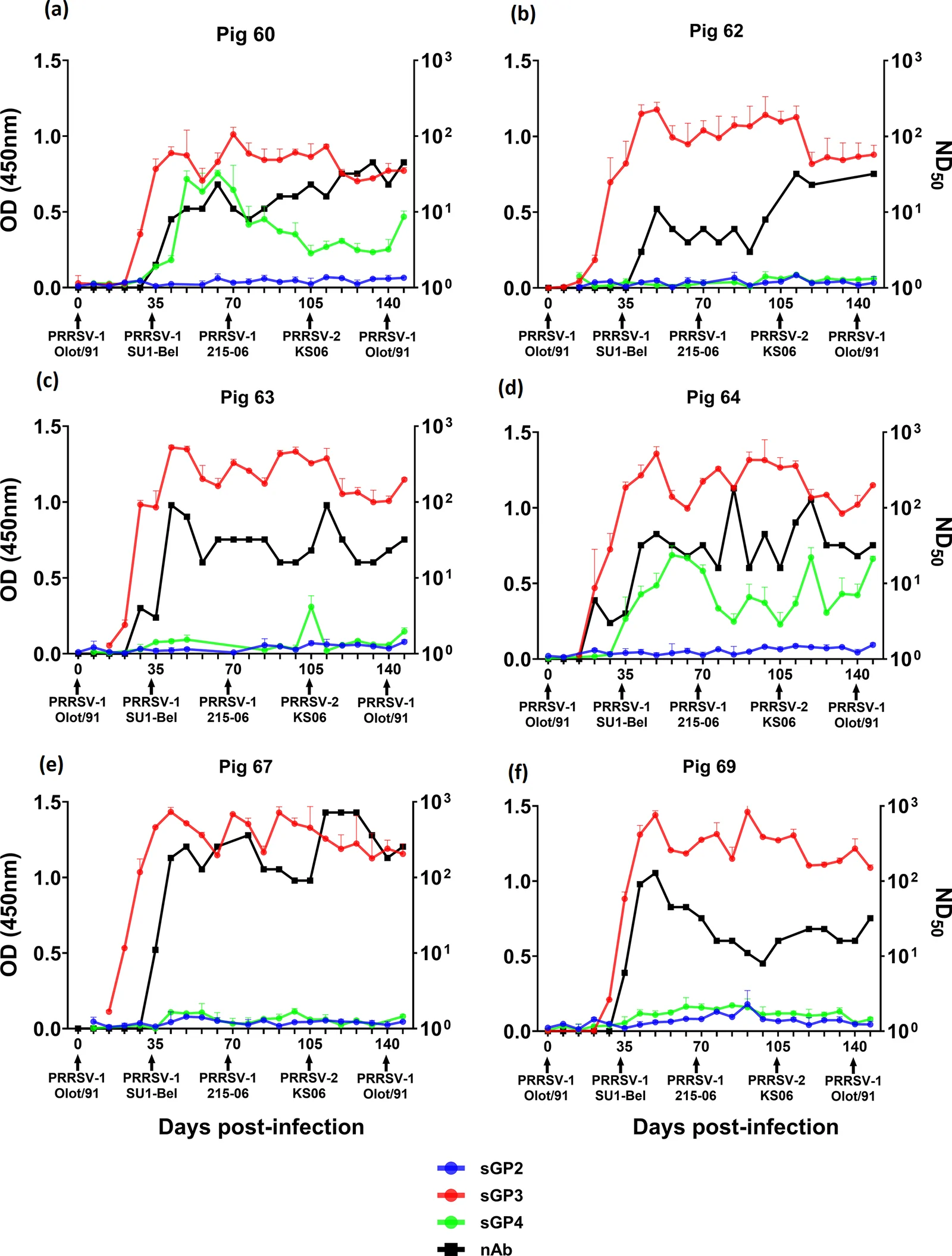
Recognition of recombinant PRRSV-1 sGP2, sGP3 and sGP4 was assessed by ELISA using sera collected from pigs (*n*=6) sequentially inoculated with diverse PRRSV strains, across a 148-day challenge study. ELISA binding responses [mean data (OD_450_) across two technical replicate wells±sd] are shown alongside independently determined PRRSV-1 Olot/91 neutralization titres (ND_50_) obtained by virus neutralization assay. All antigens were coated at 100 ng/well after normalization by BCA assay. Neutralization titres were determined independently using a virus neutralization assay with the same serum samples [56] and were compared with ELISA binding responses.

ELISAs were performed to determine recognition of sGPs by serum from pigs hyperimmunized by sequential challenge with different PRRSV-1 and -2 strains, as previously described [56] (Fig. 2a–f). The response to sGP3 was consistently the strongest throughout, arising in each pig 14–21 days post-infection (dpi), and persisting until termination of the study, with all sera from 21 to 146 dpi being significantly greater than 0 dpi serum (adjusted *P* value <0.0001). The kinetics of responses against sGP3 did not appear to be influenced by later PRRSV challenges and was a consistent trend between the six animals. In all instances, while there was a clear boosting effect following PRRSV-1 SU1-Bel challenge, this was not repeated following the subsequent challenges. The responses to sGP2 were low to negligible in pigs 60–67 across the timeline of the study, with no data points being statistically significant compared to the response at 0 dpi. An increase was observed with serum from pig 69 at 91 dpi (Fig. 2f) which was statistically significant (adjusted *P* value <0.01) although this response fell below significance by 98 dpi and remained so until termination of the study.

The responses to sGP4 differed between animals. Starting at 28 dpi and persisting until termination, serum from pig 60 and pig 64 showed significantly higher binding to sGP4 than the other animals in the cohort (adjusted *P* value <0.0001) (Fig. 2a, d), with the response from the endpoint sera remaining significantly higher than 0 dpi (adjusted *P* value <0.0001). While the response kinetics from pig 60 appeared to align with those described against sGP3, responses from pig 64 differed, with a recall effect following each challenge. No significant binding from pig 62, pig 67 or pig 69 was observed at any time point assessed (Fig. 2b, e, f), and responses from pig 63 remained below significance for most time points (Fig. 2c). The PRRSV-1 neutralizing titres of serum from each animal were compared with the antibody binding to each of the recombinant sGPs. The kinetics of the neutralizing antibody response closely mirrored the antibody reactivity to sGP3. Therefore, we hypothesized that sGP3-specific antibodies may, at least in part, contribute to the neutralizing response and were therefore our focus for the remainder of this study.

### Isolation of PRRSV-1 GP3-specific porcine mAbs

**Fig. 3.**
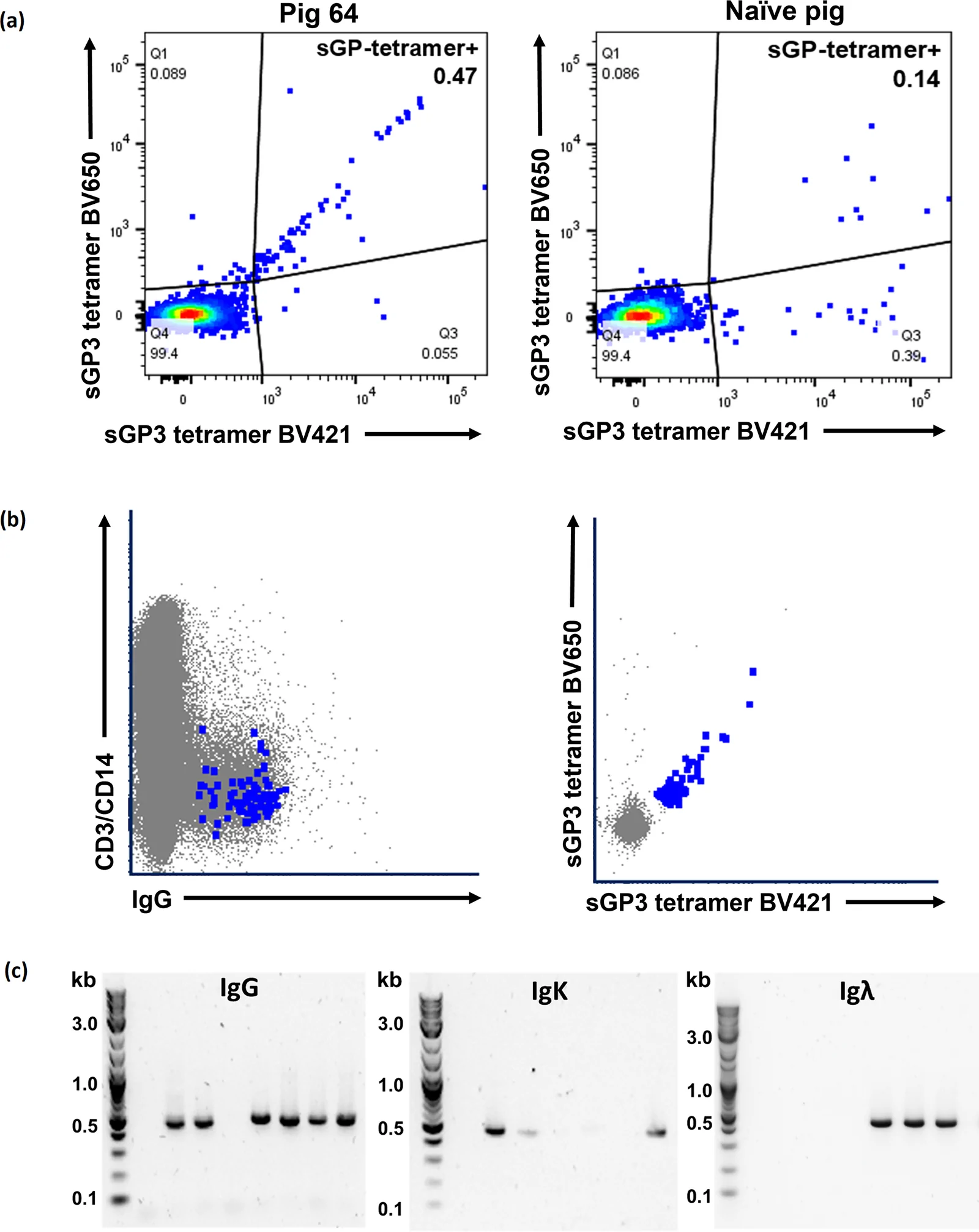
Isolation of recombinant PRRSV-1 sGP3 binding B cells from a hyperimmune pig. sGP3 tetramer labelling of IgG B cells within PBMC from hyperimmune pig 64 (148 dpi) and a PRRSV-naïve pig (**a**). Back gating of sorted single sGP3 tetramer-binding IgG B cells from pig 64 (**b**). cDNA was synthesized from the isolated single cells, and the variable region domains of IgG (VH) and Igκ and Igλ (VL) were amplified, and representative gel electrophoresis images are shown (**c**). Expected product sizes were 472 bp for VH chains, 477–512 bp for kappa chain and 422–477 bp for lambda chain.

sGP3-BV421 or -BV605 tetramers were then applied as part of an antibody panel to identify sGP3 specific IgG^+^, IgM^+^ and IgA^+^ B cells within PBMC, as previously described [42]. PBMCs cryopreserved on 148 dpi from the six pigs from the heterologous challenge were stained and analysed by flow cytometry (Fig. S6). The results showed sGP3 tetramer labelling of IgG, IgA and IgM B cells from all six challenge study animals, with a significantly higher frequency of IgG^+^ (adjusted *P* value <0.001 for all pigs), IgM^+^ (adjusted *P* value <0.05 for all pigs) and IgA^+^ (adjusted *P* value <0.05 for all pigs) sGP3 tetramer-positive events compared to a PRRSV-naïve pig. Given the high level of sGP3 reactivity and neutralizing antibody titre, pig 64 was selected for mAb isolation. A total of 110 double sGP3 tetramer-positive IgG B cells were single-cell sorted (Fig. 3a, b). The relatively low number of cells recovered likely reflects the low frequency of sGP3-specific memory B cells within the available cryopreserved PBMC sample, together with the stringent dual-tetramer gating strategy used to maximize antigen specificity and minimize recovery of non-specific B cells. Following reverse transcription, 66 (60%) of the IgG variable heavy (VH) and 44 (40%) of the light chains (VL) were successfully amplified, resulting in a total of 37 (33.64%) VH/VL pairs (Fig. 3c). Each pair was then submitted for Sanger sequencing. In total, 16 VH and VL pair sequences were identified, with several pairs possessing the same sequence, and all the heavy chains were IgG1.

The IMGT-based annotation of the antibody sequences determined the expected structure of the framework and complementary determining regions, from which we could clearly identify the CDR motifs (Table S4). Annotation of CDR motifs revealed several instances of repeated sequences: eight of the nine amplified kappa light chains shared identical (or near-identical in the case of IgLC-Κ-11D) CDR3 motif sequences, though distinct CDR1 and CDR2 motif sequences. Two instances of identical CDR2 and CDR3 sequences were identified for the lambda light chains, i.e. IgLC-λ-5E and -5F, as well as IgLC-λ-7A and -7E, with the latter also sharing identical CDR1 motifs. For the VH sequences, four sequences were seen to be completely identical, i.e. IgG1-11B, -11C, -11D and -12C, with an additional four sequences sharing identical CDR1, CDR2 and CDR3 motifs (i.e. IgG1-11E and -11F) though their FR1 motif sequences differed by one amino acid residue. Furthermore, it was determined that, although several VH sequences (i.e. IgG1-11B, -11C and -11D) were identical, their respective VL sequences were unique. The reverse was also true for IgG1-7A and IgG1-7E, which shared an identical VL sequence IgLC-λ7A|7E, despite having unique VH sequences. Only one of each repeat was selected to be taken forward in each instance, resulting in 15 unique heavy- and light-chain pairs (GenBank: PZ206307–PZ206322). A total of 7 antibodies of 15 were seen to be expressed at a level from which they could be purified, with mAbs 8–15 falling below the limit of detection by western blot. Western blotting also revealed that expression of mAbs 1, 2, 5, 6 and 7 were comparable to the control mAb, while mAbs 3 and 4 were represented only by faint bands, being close to the limit of detection (Fig. S7A). All mAbs exhibited band profiles consistent with what has been described in the literature [57] (Fig. S7B, C). Additionally, the concentration of IgG was measured in the supernatants by ELISA and found to be between 250 and 1,650 µg ml^−1^ (Fig. S8A). Despite high IgG concentrations observed, mAb 1 did not demonstrate any reactivity to sGP3 by ELISA (Fig. S8B). mAbs 2–7 all demonstrated sGP3 binding, and consequently, these six mAbs were taken forward for larger-scale Expi293F transfection and purification.

### Characterization of PRRSV-1 GP3-specific porcine mAbs

**Fig. 4.**
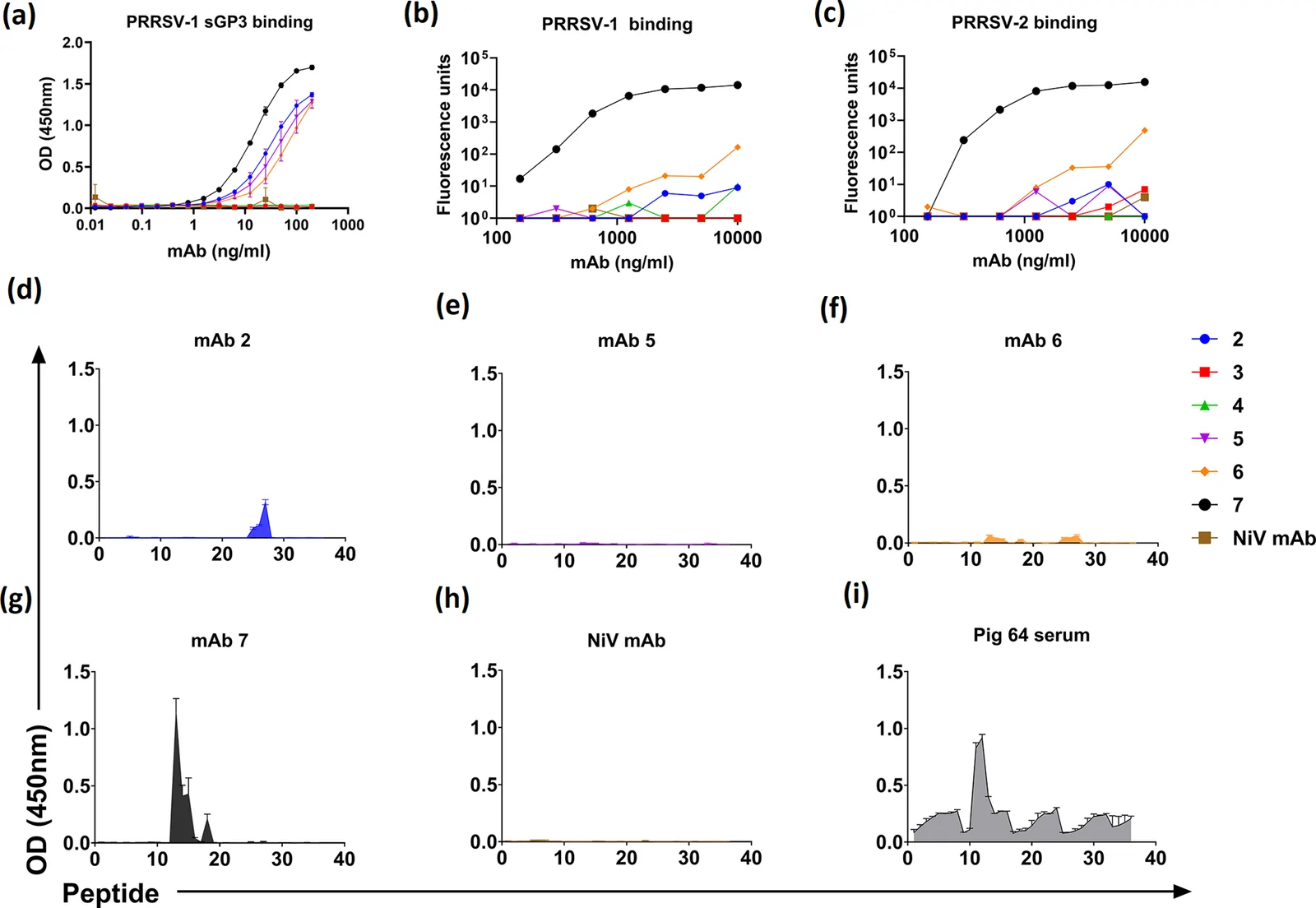
Characterization of PRRSV-1 GP3-specific porcine mAbs. Serial dilutions of purified recombinant mAbs were assessed by ELISA to determine antigen specificity (**a**). An irrelevant Nipah virus GP-specific recombinant porcine mAb (NiV mAb) was included as a negative control. Mean data of two technical replicates±sd are shown. Recognition of native PRRSV antigen was assessed by intracellular staining of MARC-145 cells infected with PRRSV-1 Olot/91 (**b**) or PRRSV-2 VR-2332 (**c**). mAb binding was revealed by the addition of a fluorescent secondary antibody and analysis using the Incucyte^®^ SX5 Live-Cell Analysis System. Technical singlets are shown. Overlapping 16-mer linear peptides spanning the PRRSV-1 GP3 ectodomain were screened for mAb recognition by ELISA (**d–i**). In addition to the four GP3-specific recombinant mAbs and the irrelevant NiV mAb, polyclonal sera from pig 64 were included. Mean data of two technical replicates±sd are shown.

Purified mAbs were assessed for sGP3 binding, the breadth of their cross-reactivity and neutralization capacity. The mAbs were first assessed by ELISA for their ability to bind the recombinant PRRSV-1 sGP3 (Fig. 4a). Compared to the control mAb, mAbs 2, 5, 6 and 7 bound to sGP3 (adjusted *P* value<0.0001). Unexpectedly, mAbs 3 and 4 did not demonstrate significant binding following scale-up and purification. mAb 7 reactivity was significantly higher than any of the other mAbs (adjusted *P* value <0.0001). mAb 2 also showed significantly higher absorbance than mAb 5 (adjusted *P* value<0.05) and mAb 6 (adjusted *P* value <0.0001), although no significant difference was observed between mAbs 5 and 6. Next, mAbs were assessed for their ability to label native GP3 in PRRSV-1- and PRRSV-2-infected MARC-145 cell monolayers (Fig. 4b, c, respectively). mAbs 6 and 7 labelled both PRRSV-1- and PRRSV-2-infected cells. mAb 2 also demonstrated binding to PRRSV-1-infected cells; however, it did not bind PRRSV-2 at the highest concentration. mAbs 3, 4 and 5 did not demonstrate any significant binding to fixed infected cells.

To further interrogate mAb specificity, a library of 16-mer, linear peptides spanning the GP3 ectodomain were screened by ELISA. Analysis was restricted to the four GP3-specific mAbs. mAb 2 showed clear binding to peptides 25–28 containing the consensus sequence VDKLH and which clearly elicited a peak observed from peptide 27. No significant binding was observed from mAb 5 to any of the peptides. Peptides 11–13 containing the consensus sequence DHDEL elicited strong binding from mAb 7, peaking at peptide 13. Significant binding could also be observed against peptide 18, containing the consensus sequence DNLKL; however, significant binding was not detected with either peptide 17 or peptide 19. mAb 6 also had two distinct binding peaks, appearing to bind similarly to mAb 2 and mAb 7 with peaks at peptide 27 and peptide 13. Although low, binding to both peptides was significant compared to the control mAb (adjusted *P* value<0.0001). The peptide mapping data, alongside the PRRSV-infected cell staining data, suggest that mAbs 2 and 7 recognize linear epitopes, whereas mAb 5 likely only recognizes a conformational epitope because it bound recombinant GP3 but not any linear peptide. Although weaker, the peptide reactivity observed for mAb 6 was substantially lower than its binding to recombinant and virus-associated antigen, suggesting recognition of a structure-dependent epitope.

Finally, the PRRSV neutralizing capability of each of the four GP3-specific mAbs was assessed. The results revealed that none of the mAbs demonstrated neutralizing activity against PRRSV-1 or PRRSV-2 (Fig. S9).

### Evaluation of the immunogenicity of recombinant PRRSV-1 GP3

**Fig. 5.**
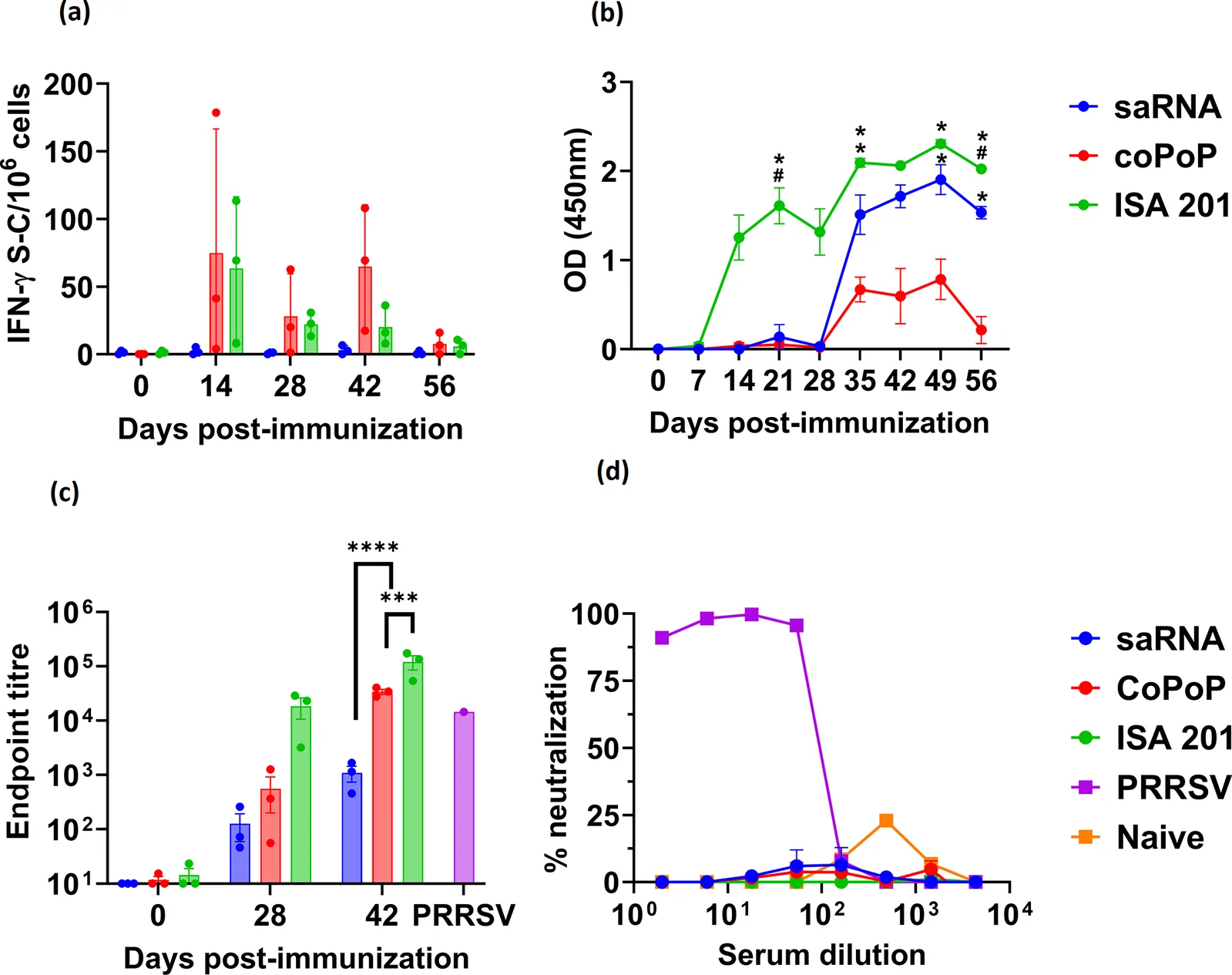
Assessment of the immunogenicity of recombinant PRRSV-1 GP3. Groups of pigs (*n*=3) were primed (day 0) and then boosted (day 28) with GP3 delivered either via an saRNA vector, on the surface of CoPoP liposomes or adjuvanted with Montanide^™^ ISA 201 VG (ISA 201). PBMCs were stimulated with GP3 peptides, and the frequency of IFN-γ secreting cells was assessed by ELISpot assay (IFN-γ S-C /10^6^ cells) (**a**). Serum was collected weekly and assessed for reactivity against recombinant sGP3 by ELISA, with statistical differences between ISA 201 and CoPoP shown by *P<0.05 and **P<0.01, and between ISA 201 and saRNA shown by ^#^*P*<0.05 (**b**), and endpoint titres for each serum were determined at days 0, 28 and 42, with statistical differences between groups shown by ***P<0.001 and ****P<0.0001 (**c**). The neutralization of PRRSV-1 was determined for each serum at day 56 and compared to a PRRSV-immune pig (PRRSV) and a naïve animal (naïve) (**d**). Mean data for each group are shown, and error bars represent sd. All statistical differences determined by 2-way ANOVA and Tukey’s multiple comparison test as shown.

The isolation of only four GP3-specific mAbs prevented us from drawing a firm conclusion regarding its role as a neutralizing antibody target. We therefore carried out a pilot immunogenicity study in pigs to assess whether GP3 elicited a neutralizing antibody response using the three vaccines: (i) an saRNA vector expressing the membrane-anchored GP3 ectodomain for cell surface expression (Fig. S1); (ii) a CoPoP formulation in which His-tagged sGP3 was inserted into the cobalt porphyrin phospholipid bilayer of adjuvanted liposomes (Fig. S2); and (iii) sGP3 delivered in ISA 201 adjuvant. GP3-specific T cell IFN-γ responses were measurable from day 14 following immunization with the saRNA-vectored GP3 and, to a lesser extent, sGP3 in ISA 201, although these responses were not statistically greater than day 0, due to inter-animal variation and the small group sizes (Fig. 5a). No significant responses were observed in the CoPoP-vaccinated group. A robust sGP3-binding antibody response was observed in the ISA 201 adjuvanted group from 14 days, which was clearly augmented at day 35 following the boost (Fig. 5b). The sGP3-binding antibody responses in the saRNA and CoPoP were reduced and could only be detected from day 35. The concentration of sGP3-binding antibodies, shown as endpoint titres, in the ISA 201 group at day 42 was significantly higher compared to days 0 (*P*<0.0001) and 28 (*P*<0.0001) (Fig. 5c), as well as the day 42 titres in the CoPoP (*P*<0.0001) and saRNA (*P*<0.001) groups. Despite sGP3-binding antibody titres being broadly comparable to those of the PRRSV-neutralizing control serum, day 56 serum samples from all three groups were unable to neutralize PRRSV-1 Olot/91 infection of MARC-145 cells (Fig. 5d) or porcine alveolar macrophages (Fig. S10). Linear epitope mapping, utilizing the overlapping GP3 peptide library, showed significant binding to peptides 11–13 by day 56 sera from all three vaccine groups, with responses again peaking at peptide 12 (Fig. S11).

### Assessment of recombinant PRRSV-1 GP3-binding antibody depletion on serum neutralization

**Fig. 6.**
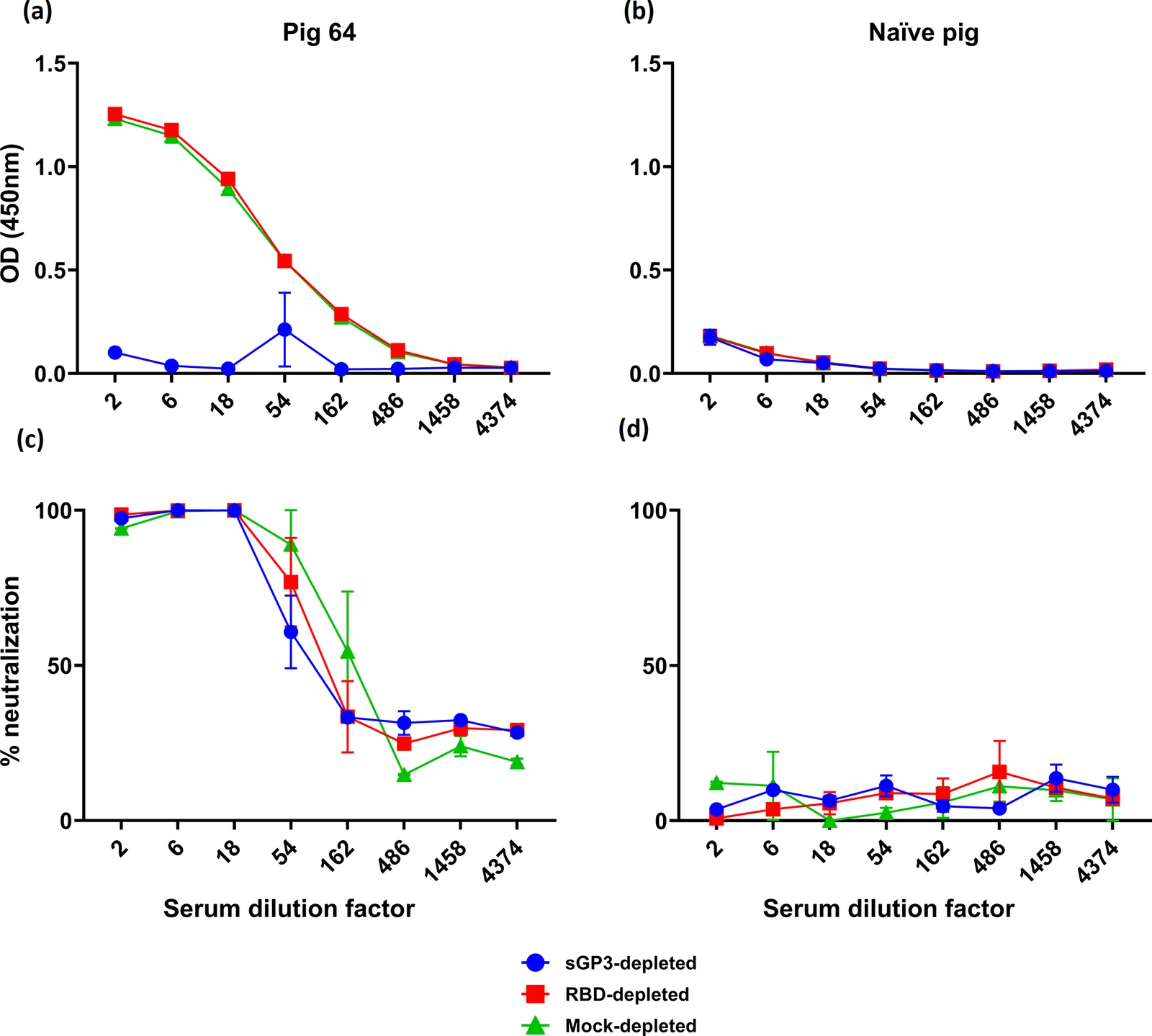
Assessment of sGP3-binding antibody depletion on PRRSV-1 neutralization. Serum from the hyperimmune pig 64 (**a, c**) and naïve pig serum (**b, d**) were each depleted under three conditions: using recombinant PRRSV-1 sGP3, recombinant SARS-CoV-2 S-RBD (to confirm assay specificity) or with a mock depletion using no antigen. The effect of antibody depletion was assessed by sGP3 ELISA (**a, b**) and VNT (**c, d**). Mean data of two technical replicates±sd are shown.

Finally, as a complementary approach, we assessed the effect of depleting sGP3-binding antibodies on serum neutralization of PRRSV-1. Serum from hyperimmune pig 64 and a PRRSV-naïve pig was taken through three separate magnetic depletions, utilizing either recombinant PRRSV-1 sGP3, recombinant S-RBD (as a non-PRRSV control antigen) or a mock depletion with no antigen present. The efficacy of sGP3-binding antibody depletion was first assessed by ELISA (Fig. 6a, b) and then VNT (Fig. 6c, d). The sGP3 binding demonstrated by the sGP3-depleted sera was significantly lower for the pig 64 sera compared to the RBD and mock-depleted sera. sGP3-depleted serum from pig 64 demonstrated reduced binding up until a 1 : 486 dilution (adjusted *P* value <0.0001). The PRRSV-naïve sera did not demonstrate any significant sGP3 binding. No significant reduction in PRRSV-1 neutralization was observed for the pig 64 serum in either protein depletion condition compared to the mock depletion. As expected, the PRRSV-naïve serum, irrespective of depletion protocol, did not demonstrate significant PRRSV-1 neutralization.

## Discussion

PRRSV are rapidly evolving and diversifying with novel highly pathogenic strains emerging from both species [58–60]. Despite decades-long availability of both MLV and inactivated vaccines, protection is often insufficient, conferring little to no protection against more genetically heterologous strains [25]. There is, therefore, an urgent requirement to explore alternative approaches to PRRSV vaccine development. The PRRSV minor envelope complex GPs GP2, GP3 and GP4 are essential for viral entry and have all been implicated in containing neutralizing antibody epitopes [19, 21, 61, 62]. To better understand the antigenic properties of these individual GPs and their potential contribution to neutralizing antibody responses, we used mammalian cells to express recombinant soluble PRRSV-1 GP2, GP3 and GP4 ectodomains and assessed their recognition by antibodies from pigs hyperimmunized by sequential heterologous PRRSV challenge infection [56]. Evaluation of recombinant GP expression revealed consistently higher levels of sGP3 secreted into culture supernatants compared to sGP2 and sGP4. It has been previously described that, as a result of its unique hairpin-like topology, a soluble form of PRRSV GP3 is secreted from infected cells [63]. This may contribute to the difference in secretion observed, particularly following the removal of the predicted transmembrane domain. After purification, the GP banding patterns became less distinct with the glycoforms appearing as one broad band. Digestion by PNGase F revealed a single band confirming multiple glycoforms. GP2, GP3 and GP4 are known to possess two, seven and four potential N-glycosylation sites, respectively [54, 64], which are conserved across both PRRSV-1 and -2 strains. Variations in glycan types and glycosylation of proteins are a major limiting factor in recombinant antigen production, as glycosylation can dramatically impact vaccine efficacy. This level of glycosylation may also have contributed to the low level of expression of these GPs in mammalian cells [65]. Mass photometry analysis indicated that sGP3 predominantly existed as a dimeric species in solution, with limited evidence of higher-order aggregation, although interpretation is complicated by heterogeneous N-linked glycosylation. Analysis of the recombinant sGP3 sequence using the NetNGlyc 1.0 server predicted four high-confidence N-glycosylation sites, consistent with the multiple glycoforms observed by SDS-PAGE and PNGase F digestion. Whether this oligomeric state influences antigenicity or neutralizing epitope presentation remains unclear. Additional bands observed following purification may have also represented host-cell proteins co-purified during IMAC purification. Although these impurities could potentially influence polyclonal serum analyses, the successful isolation of GP3-specific mAbs supports the presence of authentic GP3 epitopes within the preparation. Additional polishing steps such as size-exclusion chromatography may further improve antigen purity in future studies.

Compared to recombinant PRRSV-1 sGP2 and sGP4, sGP3 consistently showed a higher level of reactivity with antibodies within the serum from PRRSV hyperimmunized pigs. These data suggest that PRRSV-1 GP3 elicits a higher antibody response than GP2 and GP4 in this experimental setting, which is consistent with the high number of antigenic regions described for this GP [66]. The lower and inconsistent antibody reactivity against PRRSV-1 sGP2 and sGP4 was perhaps surprising compared to what has been described in other studies using synthetic peptides [66–69]. Though PRRSV-1 GP4 possesses a highly antigenic neutralizing epitope, antibody responses against this linear epitope were only measurable in a proportion of infected pigs [67, 68]. It is well documented that GP4 contains epitopes within a hypervariable portion of its sequence [67, 70], most likely a driver of immune escape mutations, and so antibody responses against this epitope may not have been boosted or sustained by the subsequent challenges with heterologous PRRSV strains.

The robust antibody response of the hyperimmunized pigs to PRRSV-1 sGP3 and the comparable kinetics to the PRRSV neutralizing antibody response led us to hypothesize that this protein bore neutralizing epitopes, as corroborated by the literature [66]. However, subsequent analyses failed to provide evidence to support this hypothesis. Neither sGP3-specific mAbs isolated from the hyperimmune pigs, which recognized distinct epitopes, nor polyclonal serum from pigs immunized with sGP3 using vaccine formulations that elicited high titre antibody responses were able to neutralize PRRSV-1. One explanation for the lack of neutralization might be that GP3 is well documented for possessing non-neutralizing antigenic regions [71, 72], serving as decoy epitopes and directing B cell responses away from epitopes which would neutralize PRRSV. The non-neutralizing mAb 7 showed cross-reactivity against PRRSV-1 and PRRSV-2, and linear epitope mapping suggested binding to the peptide sequence DHDEL. This sequence is relatively well conserved between PRRSV-1 and PRRSV-2 and has been proposed by others as a highly antigenic decoy epitope in the context of PRRSV-2 [73]. Similarly, the non-neutralizing mAb 2 reactivity was mapped to the GP3 peptide VDKLH, which is a documented epitope in the context of the PRRSV-1 Lelystad strain, shown to be immunogenic but non-neutralizing [71]. These findings align with a recent study that found no correlation between antibody reactivity to linear epitopes on PRRSV-1 envelope GP ectodomains and virus neutralization [71]. However, these data differ from a previous study where GP3 peptide-specific antibodies showed neutralization against PRRSV-1 Lelystad [66]. Differences in antigen design (peptide vs. ectodomain) and expression system may lead to conformational differences that account for this discrepancy. A notable example is the mAb PN9cx3, which was isolated from PRRSV-2 GP3-immunized mice and was shown to broadly neutralize diverse PRRSV-1 and PRRSV-2 strains and confer protection against challenge in piglets following passive transfer [61]. In contrast, none of the recombinant sGP3-binding mAbs isolated in the present study demonstrated measurable neutralizing activity. Differences in antigen presentation may have influenced the specificity of the mAbs isolated. Zhang *et al.* generated PN9cx3 following immunization with insect cells infected with recombinant baculovirus expressing PRRSV-2 GP3, whereas our study utilized a soluble mammalian cell-expressed PRRSV-1 GP3 ectodomain as both the antigen bait for B cell sorting and as a vaccine immunogen. Interestingly, PN9cx3 was reported not to recognize GP3 recombinantly expressed in either insect or mammalian cells, leading the authors to speculate that differences in glycosylation or antigen conformation may influence presentation of a conserved neutralizing epitope [60]. These observations highlight a potential limitation of studies employing recombinant GPs and suggest that critical neutralizing epitopes may only be formed or exposed in specific structural contexts.

Furthermore, a caveat to the present study was the small number of GP3-specific mAbs generated. Broadly neutralizing GP3-specific antibodies may represent a comparatively rare population that was not captured within the limited number of B cells and antibody clones recovered in this study. This was likely influenced by the relatively low frequency of recoverable sGP3-specific B cells present within the available cryopreserved PBMC samples. While encouraging that a proportion of the isolated mAbs were able to recognize native antigen, they are unlikely to represent the full B cell repertoire specific to GP3. Future studies using larger B cell repertoires or alternative antigen baits may help overcome this limitation.

Importantly, the interpretations of our study are limited by the use of a soluble GP3 ectodomain rather than the native GP2–GP3–GP4 complex present on virions. While secretion of a soluble and glycosylated protein is consistent with productive folding, it does not guarantee preservation of all conformational epitopes present in the native envelope complex. A study investigating the immunogenicity of adenovirus-expressed PRRSV-2 GP3 in mice found that immunization with a truncated version of GP3 (deletion of amino acids 2–64) elicited stronger neutralizing antibody and cellular immune responses than the full-length protein, suggesting that GP3 conformation and epitope presentation can substantially influence immunogenicity [74]. While the relevance of these findings to protection in pigs remains unclear, they support the possibility that alternative GP3 constructs may expose antigenic determinants not adequately represented by the isolated ectodomain used in the present study. It remains possible that additional, potentially neutralizing conformational epitopes are present outside the ectodomain or are formed only in the context of full-length GP3 and its interaction with GP2 and GP4. Future studies should consider GP3 in combination with GP2 and GP4 or use complete full-length constructs, including transmembrane regions and natural glycosylation to better define its role in neutralization.

Evaluation of GP3 as a vaccine candidate antigen has typically used the full-length GP and often in combination with other PRRSV GPs, particularly GP5. For example, a recombinant adenovirus vector co-expressing full-length PRRSV-1 GP3 and GP5 induced neutralizing antibodies and provided pigs with partial protection [75]. Immunization of mice with plasmid DNA vaccines co-expressing PRRSV-1 or -2 GP3 and GP5 appeared to have a synergistic effect, with enhanced neutralizing antibody responses compared to plasmids expressing the individual GPs, which were weak in mice immunized with GP3 alone [76, 77]. This synergistic effect has also been observed in an early subunit vaccine study, where improved protection was observed when pregnant sows were immunized with a combination of insect-cell expressed PRRSV-1 GP3 and GP5 [78]. Importantly, however, each of these studies assessed neutralization only against homologous strains or was limited to the prototype PRRSV-1 Lelystad strain only, and evidence for induction of neutralizing Abs capable of broad protection is lacking. saRNA platforms, like that detailed in this study, offer a promising approach for delivering PRRSV antigens such as GP3 in combination with other viral proteins, owing to their high protein expression efficiency, dose-sparing potential and ability to mimic natural viral infection. This is particularly advantageous for veterinary vaccines, where cost and scalability are critical. Co-expressing GP3 with the other minor envelope GPs GP2 and GP4 or other candidate antigens such as GP5 and M via saRNA could synergistically enhance both humoral and cellular immune responses. Co-delivery of these proteins on a single saRNA construct could allow for coordinated antigen expression, promoting epitope spreading and stronger immune priming.

Though the results of the depletion experiment may seem compelling evidence that sGP3-binding antibodies do not contribute to neutralization, the absence of an effect does not definitively exclude a minor role for GP3 ectodomain-directed neutralization. It has also been hypothesized that a multi-antigenic response against the minor envelope proteins may contribute to serum neutralization [79]. Our findings support the view that serum neutralization of PRRSV may be reflective of polyclonal antibody responses targeting multiple structural proteins rather than a single dominant antigen.

In summary, this study has shown that recombinantly expressed PRRSV-1 GP3 ectodomain is antigenic, possessing conserved antibody epitopes recognized by immune pigs. However, when delivered as an immunogen, it did not elicit a measurable neutralizing antibody response. This distinction between immunogenicity and neutralizing potential is relevant for PRRSV vaccinology and provides groundwork for subsequent research into PRRSV minor envelope GPs, and the role they play in the immune response against PRRSV, information that will be key in the development of the next-generation vaccines that are urgently required to improve PRRSV control.

## Supplementary material

10.1099/jgv.0.002328 Supplementary Material 1.

## Abbreviations

AEC: 3-amino-9-ethylcarbazole
APHA: Animal and Plant Health Agency
blast: Basic Local Alignment Search Tool
bnAbs: broadly neutralizing antibodies
BV421: Brilliant Violet 421
CDRs: complementarity-determining regions
CoPoP: cobalt porphyrin-phospholipid
dpi: days post-infection
GPs: glycoproteins
HEK: human embryonic kidney
IFN-γ: IFN gamma
IMGT: ImMunoGeneTics information system
LNPs: lipid nanoparticles
mAbs: monoclonal antibodies
MLV: modified live virus
PBMCs: peripheral blood mononuclear cells
PRRSVs: porcine reproductive and respiratory syndrome viruses
RBD: receptor-binding domain
RT: room temperature
RT-PCR: reverse transcription polymerase chain reaction
saRNA: self-amplifying ribonucleic acid
sgmRNA: subgenomic mRNA
VNT: virus neutralization test
