# Supplementary material for: Evaluation of the contribution of the minor envelope complex glycoprotein 3 ectodomain to the porcine reproductive and respiratory syndrome virus 1 neutralizing antibody response
Source: J Gen Virol. 2026 Sep 3;107(9):002328. doi: 10.1099/jgv.0.002328 (PMC13541077; doi:10.1099/jgv.0.002328)
Supplement: Supplementary file 1 [file jgv-107-02328-s001.pdf]

A

ATGAAGTCGATCCAGCTTTGCATCCTACTCTGGTGCTTGAGAGCAGTCTGCTG
CCATTCAAATTCCAATTCAACTCTGTGTTTTTGGTTTCCGCTCGCACACGGCAA
TACCTCTTTTGAGCTTACCATTAATTACACAATATGCATGCCTTGCAGTACCTCC
CAGGCGGCTCACCAGAGACTGGAGCCTGGGAGAAATATGTGGTGTCGCATC
GGACATGATCGCTGTGAAGAGAGGGACCACGACGAACTTTCCATGTCCATCC
CCTCCGGGTATGACAATCTCAAATTGGAGGGATACTATGCATGGTTGGCTTTC
CTTTCCTTTTCCTACGCTGCGCAGTTTCATCCAGAGCTCTTTGGGATCGGAAA
CGTGTCCAGGGTGTTCGTGGACAAACAACATCAGTTTATTTGTGCAGAGCAC
GATGGGCGAAACTCTACGATATCAACTGAATACAATATTAGTGCGCTGTACGCA
GTGTATTATCATCACCAGATTGATGGCGGTAACTGGTTTCATCACCATCACCAT
CACTAA

B

ATGAAATCCATCCAACTTTGCATACTTCTCTGGTGTCTCAGAGCAGTCTGTTGC
CATTCCAACTCTAACTCTACGCTTTGTTTCTGGTTCCCTCTCGCTCATGGTAAT
ACTAGCTTCGAGCTGACCATCAATTATACCATTTGTATGCCCTGTAGCACGAGT
CAAGCTGCCCACCAGCGGCTGGAGCCGGGGAGAAACATGTGGTGTAGAATA
GGTCACGATCGCTGCGAGGAAAGGGATCACGATGAATTGTCTATGTCCATTCC
ATCTGGATATGATAATCTGAAGCTGGAAGGATACTATGCCTGGCTTGCCTTTTT
GTCCTTTTCTTACGCAGCTCAGTTCCACCCAGAGCTTTTTGGCATTGGTAACG
TGTCCCGCGTTTTCGTGGACAAGCAACACCAATTCATTTGTGCAGAACATGAC
GGCCGCAATTCAACCATTTCCACTGAATACAATATCTCCGCTCTGTACGCAGTT
TATTATCATCATCAGATTGATGGTGGAAACTGGTTTTGGTGGACCGGCTGGAG
GCAGTGGATTCCAGCAGGGATAGGGGTTACATTGGTTATTATCCTTGTTATTGC
TCTGTTTTGCATTTGTAAGTTTGTCTTCTAA

Figure S1: DNA sequences of PRRSV GP3 used for saRNA vaccine design. Initial constructs were designed to generate soluble protein in HEK293 cells (A) with an added His-tag highlighted in yellow. Vaccine constructs (B) were designed to include the EBOV transmembrane sequence, highlighted in purple, along with the secretion signal sequence from rat follicle stimulating hormone beta-subunit (GenBank Accession number: M36804.1) highlighted in green. Both sequences have been codon optimized for *Sus scrofa*. Stop codons are highlighted in red.

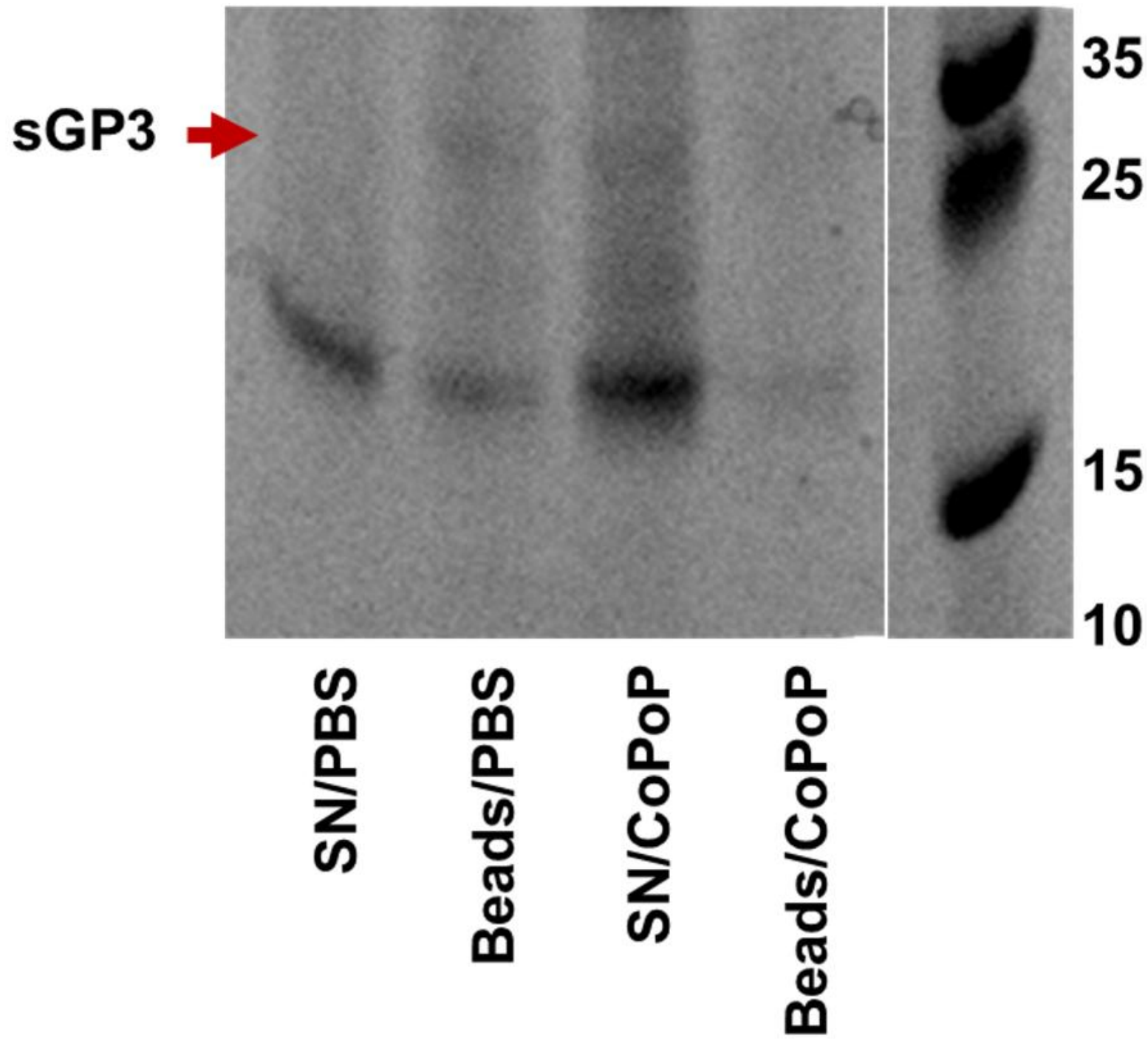

Figure S2: CoPoP liposome formation was assessed by Coomassie staining following native-PAGE. Following de-glycosylation of sGP3, association with Ni-NTA beads or the CoPoP liposome was assessed by SDS-PAGE and Coomassie staining. 1 µg of sGP3 was added to each condition. Association was determined by comparing flowthrough supernatant (SN) or Ni-NTA beads following mixture with either PBS or the CoPoP liposomes.

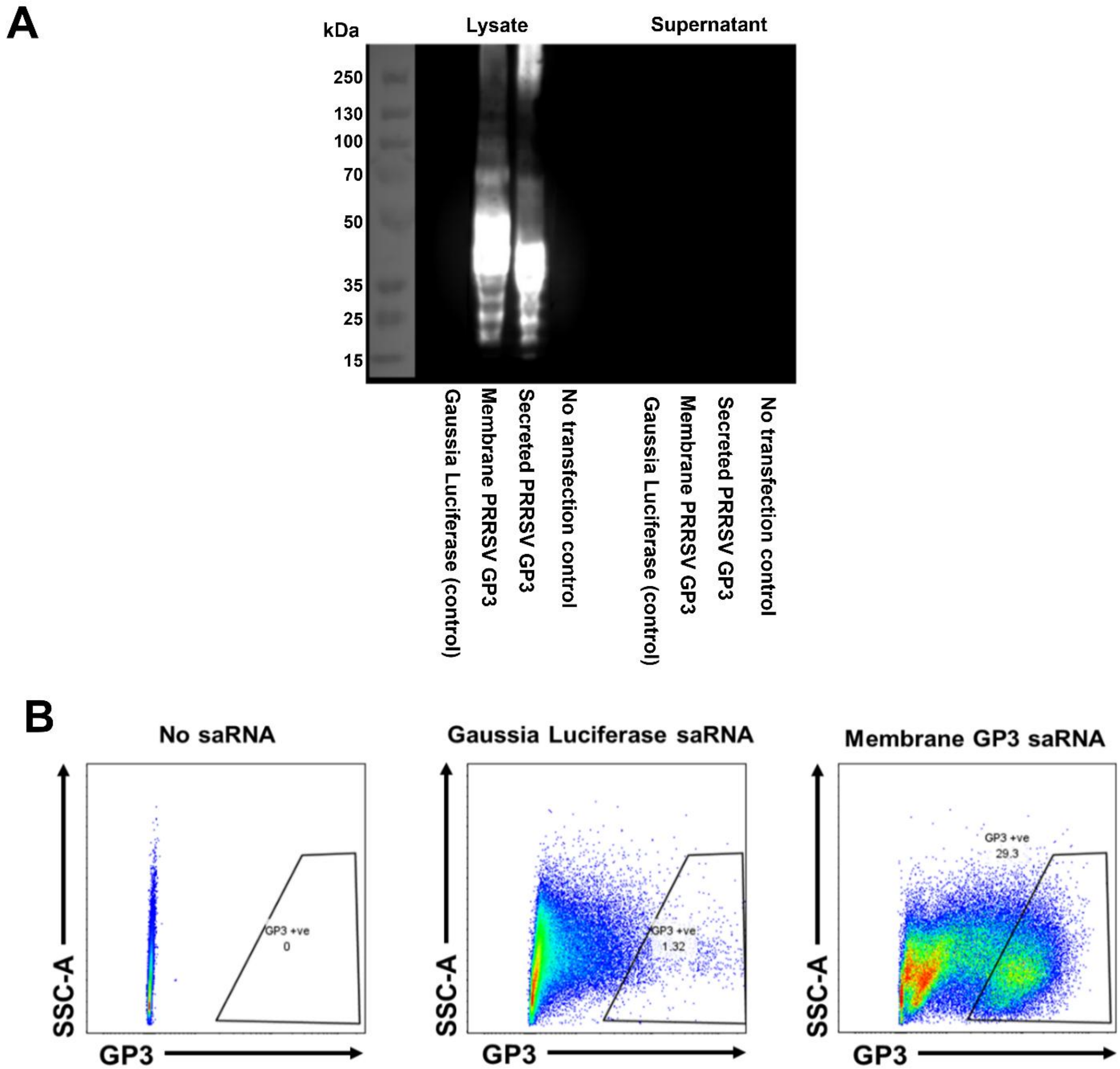

Figure S3: saRNA-vectored GP3 expression was assessed in vitro in transduced HEK293T cells. Expression was assessed by use of an anti-GP3 mAb by western blot analysis of cell lysate (A), or through flow cytometric staining (B). Cells transduced with saRNA expressing Gaussia luciferase saRNA were used in both assays as controls.

	Gene nucleotide sequence
GP2	ATGCAATGGGGTCACTGTGGCGCTAAAAGCGCATCCTGTAGCTGGACTCCTTCTTTGT<br>CATCTTTGTTGGTCTGGTTGACGCTCAGCTTCAGCTTGCCATATTGTCTTGGCTCACCA<br>TTGCCAGCTGGATACTGGTCATTCTTTTCCGAGTGGTTCGCACCTCGCTTTAGCGTCA<br>GGGCACTTCCGTTCACTCTGCCGAATTACCGGCGGTCCTACGAAGGCCTTCTGCCCA<br>ACTGTCGCCCCGACGTGCCCCAGTTCGCGGTGAAACATCCGCTTGGAATGTTTTGGC<br>ACATGAGGGTAAGTCACCTGATAGATGAGATGGTTTCTCGCCGGATATATCAAACTATG<br>GAACACTCTGGTCAAGCGGCTTGGAAACAAGTGGTATCCGAGGCGACACTGACTAAA<br>TTGAGCGGCCTCGATATAGTGACTCACTTCCAGCACTTGGCTGCCGTGGAAGCCGAC<br>AGTTGCAGATTTCTGTCCTCCAGGCTCGTCATGCTGAAAAACCTTGCGGTTGGAAATG<br>TTAGCTTGCAGTACAACACTACACTCGACAGGGTGGAGCTGATATTTCCAACTCCAGG<br>TACCCGCCCAAAACTCACAGACTTCAGACAATGGTTGATTAGTGTTCACGCCAGTATAT<br>TTTCAAGTGTAGCGAGCAGCGTTACCCTGTTCATCGTCCTTTGGCTTCGCATTCCGATA<br>CTTCGCTACGTGTTCGGTTTTCATTGGCCTACAGCTACGCATCATTCCTCCTAA
GP3	ATGGCTCATCAGTGTGCGCGATTTCATTTCTTCTTGTGCTCATTCATCTGCTACTTGGTA<br>CATTCCGCGTTGGCCTCAAATTCCAATTCAACTCTGTGTTTTTGGTTTCCGCTCGCACA<br>CGGCAATACCTCTTTTGAGCTTACCATTAATTACACAATATGCATGCCTTGCAGTACCTC<br>CCAGGCGGCTCACCAGAGACTGGAGCCTGGGAGAAATATGTGGTGTCGCATCGGAC<br>ATGATCGCTGTGAAGAGAGGGACCACGACGAACTTTCCATGTCCATCCCCTCCGGGT<br>ATGACAATCTCAAATTGGAGGGATACTATGCATGGTTGGCTTTCCTTTCCTTTTCCTACG<br>CTGCGCAGTTTCATCCAGAGCTCTTTGGGATCGGAAACGTGTCCAGGGTGTTCGTGG<br>ACAAACAACATCAGTTTATTTGTGCAGAGCACGATGGGCGAAACTCTACGATATCAACT<br>GAATACAATATTAGTGCGCTGTACGCAGTGTATTATCATCACCAGATTGATGGCGGTAAC<br>TGGTTTCATCTGGAATGGCTCCGGCCCTTTTTCTCCTCTTGGCTGGTACTTAACATATC<br>TTGGTTCCTCCGGCGGTCTCCCGTCTCACCAGTATCAAGAAGGATTTACCAGATTCTTA<br>GGCCTACGAGGCCGCGCCTCCCCGTAAGCTGGTCATTTAGAACAAGCATAGTCTCCG<br>ATCTCACTGGCAGTCAACAACGCAAGAGGACATTCCCATCAGGATCAAGATTGAATGT<br>CGTAAAACCCAGCGTGTTCCCGTCCACTCTTAG ATAA
GP4	ATGGCGGCGGCGATACTCTTTCTGTTGGCGGGAGCCCAGCATTTTATGGTATCTGAGG<br>CATTTGCCTGTAAGCCGTGTTTCTCTACTCACCTTTCTGATATTAAGACTAACACGACC<br>GCCGCGGCAGGCTTCATGGTCTTGCAGAATATTAATTGTCTTAGGCCGCACGGAGTCA<br>GCACAGCTCAAGAGAACATATCATTCGGAAAGCCCTCACAGTGCAGGGAAGCCGTAG<br>GGATACCTCAATATATAACCATTACCGCAAATGTTACCGACGAATCATATTTGTATAACGC<br>GGACCTTCTGATGCTGTCAGCGTGTCTCTTTTACGCATCCGAAATGTCAGAGAAGGGC<br>TTCAAAGTAATCTTTGGTAATGTTAGTGGAGTAGTTAGTGCGTGTGTGAATTTCACTGAT<br>TACGTGGCACATGTAACCCAGCATACTCAGCAGCACCACCTTGTGATAGACCATATCAG<br>GCTGCTTCACTTCTTGACCCCATCAACAATGCGCTGGGCAACTACCATCGCCTGCCTG<br>TTTGCGATCCTGTTGGCTATTTGA

Table S1: Human codon-optimized PRRSV-1 Olot/91 GP2, GP3, and GP4 sequences, to be used as templates to generate recombinant proteins. Start codons are in green, stop codons in red.

Primer name	Sequence (5' to 3')	Target sequence
OPPF22383-fwd	gcgtagctgaaaccggcCCATTGCCAGCTGGATACTG	5' of nucleotide position 117 of GP2 (predicted signal peptide removed)
OPPF22387-fwd	gcgtagctgaaaccggcAATTCCAATTCAACTCTGTGTTTTTG	5' of nucleotide position 81 of GP3 (predicted signal peptide removed)
OPPF22391-fwd	gcgtagctgaaaccggcGCCTGTAAGCCGTGTTTCTC	5' of nucleotide position 66 of GP4 (predicted signal peptide removed)
OPPF22383-BAP-rev	gtcattcagcaagctTTGTCTGAAGTCTGTGAGTTTTG	3' of GP2 nucleotide position 603 for addition of an AviTag
OPPF22387-BAP-rev	gtcattcagcaagctTTCCAGATGAAACCAGTTACCGC	3' of GP3 nucleotide position 543 for addition of an AviTag
OPPF22391-BAP-rev	gtcattcagcaagctCCTGATATGGTCTATCACAAGG	3' of GP4 nucleotide position 571 for addition of an AviTag

Table S2: Primers designed for the generation of recombinant PRRSV-1 sGP2, sGP3, and sGP4. All sequences were designed to enable alternative signal peptide use. Lower case nucleotides represent the overhang sequences, designed to enable cloning into the respective vectors.

Primer name	Sequence (5' to 3')	Target sequence
235	AACTGGGTGGTCTTGTTTGC	ssIGHV_L1_F
236	GGAGTAGAGCCCTGACGG	ssIGHG-R
246	GCCTCYTGCTGCTCTGG	IGKV1-F
247	TTCCCTGCTCAGCTCCTG	IGKV2-F
248	GACCACCCCATCCACTTTC	IGKC-R
251	CCTTGTCACCCTCCTCACTC	IGLV2-F
252	CTGGAYCCCTCTCCTGCTC	IGLV3-F
253	CCTGGACGGTGCTTCTGAT	IGLV8-F
300	GCGTCACTGTCTTCTCCACA	ssCL4_R

Table S3: Primers for amplification of porcine VH and VL genes.

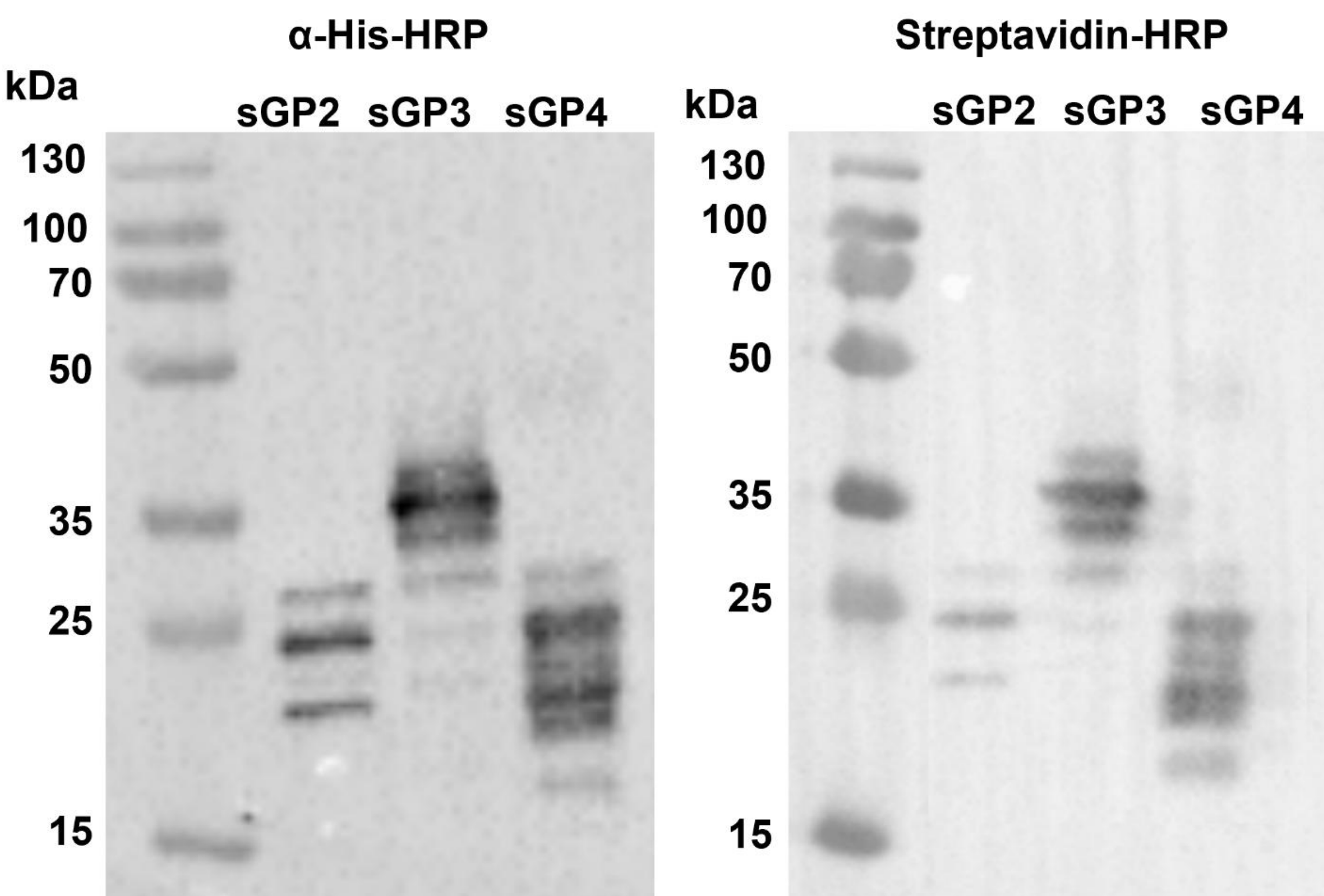

Figure S4: In vivo biotinylation of recombinant PRRSV glycoprotein ectodomains following co-transfection of HEK293T cells with BirA ligase was assessed by western blot. Protein from whole cell lysate was assessed by probing with anti-His Tag-HRP (left) or streptavidin-HRP (right).

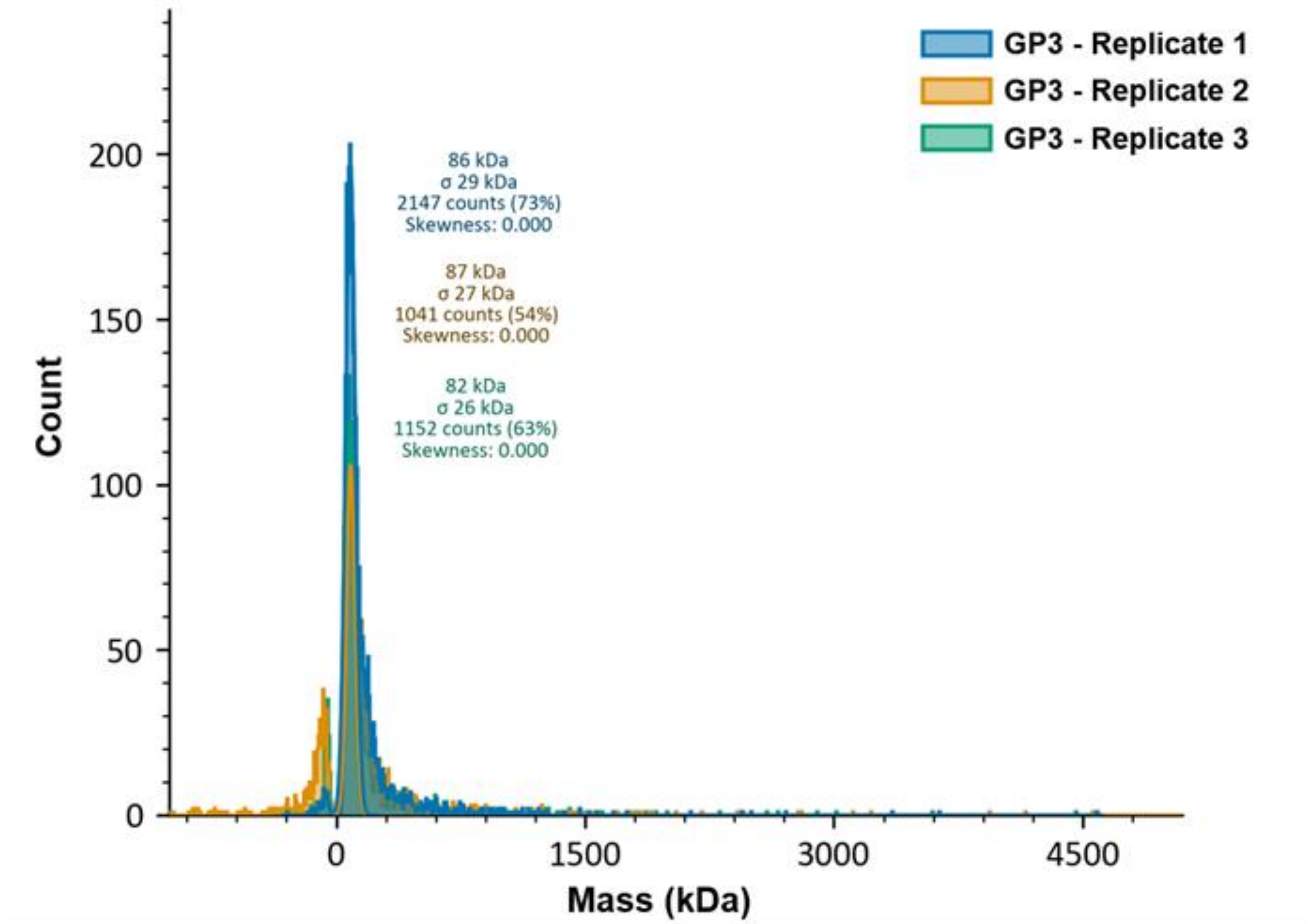

Figure S5: Mass photometry analysis of recombinant PRRSV-1 soluble GP3 (sGP3). Recombinant PRRSV-1 sGP3 was diluted to 20 nM in filtered PBS and analyzed by mass photometry. Measurements were performed in triplicate following calibration of the instrument using bovine serum albumin, β-amylase and thyroglobulin protein standards. Data shown are representative of three independent measurements.

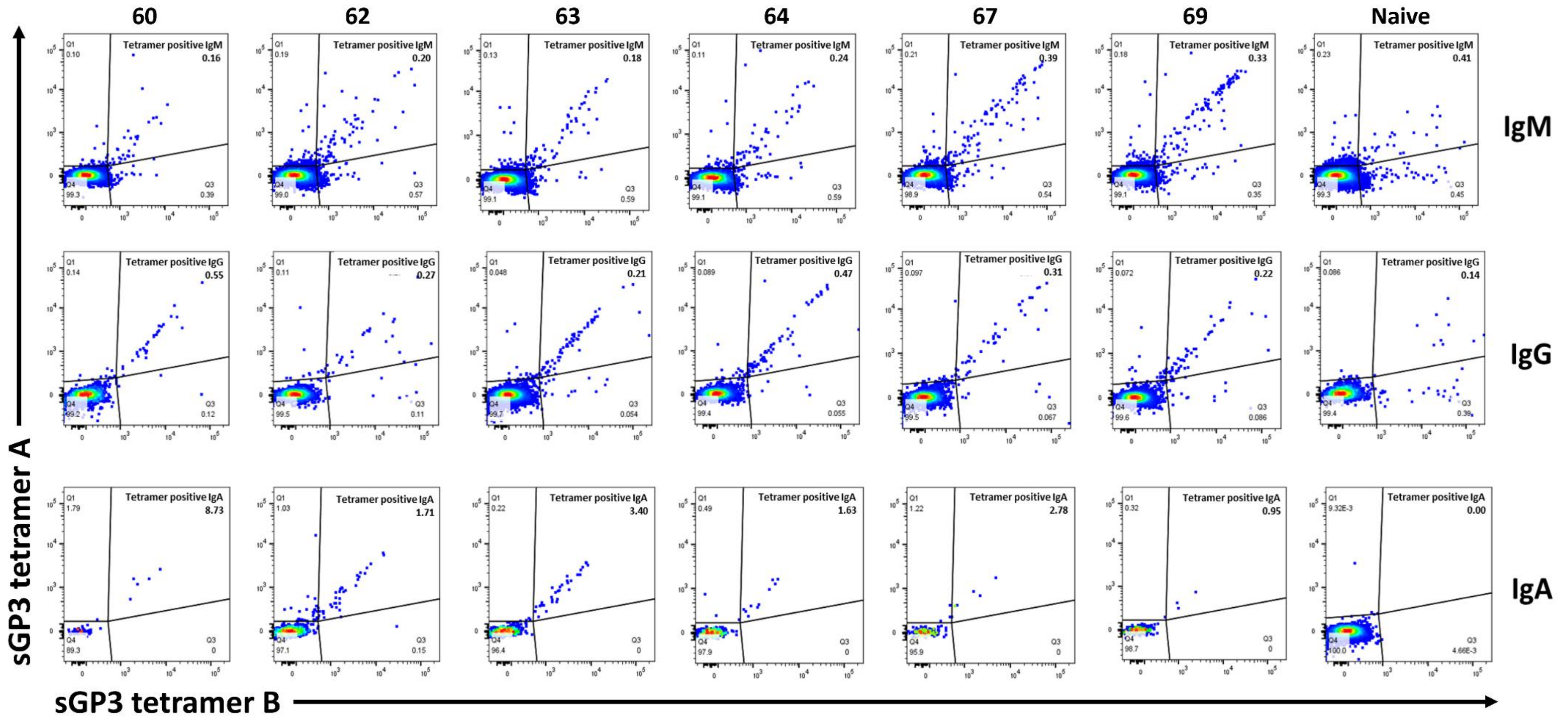

Figure S6: Flow cytometric dot plots of recombinant sGP tetramer-stained IgG, IgM and IgA expressing B cells. PBMCs from PRRSV-immune pigs (n = 6, 60-69) are compared to a PRRSV-naïve pig (naïve). Cells binding to the decoy tetramer, assembled using a non-PRRSV antigen, have been excluded. Cells in Q1 and Q3 (top left and bottom right respectively) are also excluded, with those in Q2 (top right) being considered true binders to the recombinant sGP3.

	FR1-IMGT (1-26)	CDR1-IMGT (27-38)	FR2-IMGT (39-55)	CDR2-IMGT (56-65)	FR3-IMGT (66-104)	CDR3-IMGT (105-117)	FR4-IMGT (118-128)	Closest germline	% identity
**IgG1-2G**	**EEKLVESGG.GLVQPGGSLRLTCVGS**	**GFDF....SDNS**	**FSWVRQAPGKGLEWVAA**	**IATGDYDGGT**	**FYTDSVK.GRFTISSDSSQNTAYLQMNTLRAEDTARYYC**	**ATTPRLTIAVAIAVIGVAGYRPVMDL**	**WGPGVEVVVSS**	**IGHV1-6*01**	**94.56**
IgG1-4D	EVKLVESGG.GLVQPGGSLRLSCVGS	GFTF....SSTY	INWVRQAPGKGLEWLAT	LTSS..GSVT	NYADSVK.GRSDISRDNSQNTAWLQMNSLRTEDTARYYC	AIGYIEGSATYYGMDL	WGPGVQVVVSS	IGHV1-10*01	92.01
**IgG1-5E**	**EVKLVESGG.GLVQPGGSLTLSCVGS**	**GFTF....SSIY**	**INWVRQAPGKGLEWLAA**	**ISNS..GSNT**	**YYADSVK.GRFTISRDNSQNTAYVRVNSLRTEETARYYC**	**ARCFGYGGGCYAGFYVMDL**	**WGPGVEVVVSS**	**IGHV1-4*02**	**93.75**
**IgG1-5F**	**EVKLVESGG.GLVQPGGSVRLSCVGS**	**GITF....TNTY**	**INWVRQAPGKGLGWLAT**	**MTTV..GGVT**	**NYADSVK.GRFTISRDNSQNTAYLQMNSLRTKDTARYYC**	**AIGYTGASATYYGMDL**	**WGPGVEVVVSS**	**IGHV1-10*01**	**93.4**
**IgG1-7A**	**EVKLVESGG.GLVQPGGSVRLSCVGS**	**GFTF....SSTY**	**INWVRQAPGKGLEWLAT**	**LTSS..ASVT**	**NYADSVK.GRFTISRDNSQNTAYLQMNSLRTEDTARYYC**	**AIGYVGGSATYYGMDL**	**WGPGVEVVVSS**	**IGHV1-10*01**	**90.97**
**IgG1-7E**	**EVKLVESGG.GLVQPGGSLRLSCVGS**	**GFTF....SSDF**	**MSWVRQAPGKGLEWLAC**	**ISNS..GDNT**	**YYADSVK.GRFTMSRDNSQNTAYLQMSSLKTEDTALVYC**	**VRGCGGHALDL**	**WGPGVEVVVSS**	**IGHV1-4*02**	**90.97**
**IgG1-9D**	**EVKLVESGG.GLVQVGGSLRLSCVGS**	**GISF....SIYS**	**VNWVRQGPGKGLEWLGS**	**IGRS..GTNT**	**WYADSVK.GRFTISRDNSQNTAYLQMTSLRTEDTARYWC**	**EGSCSNCPSPSTVNL**	**WGPGVEVVISS**	**IGHV1-8*01**	**89.58**
IgG1-11A	EEKLVESGG.GLVQPGGSLRLSCVGS	GFTF....STYE	IIWVRQAPGKGLEWLAA	ISTR..GSSF	DYADSVK.GRFTINRDDSHNTATLQMNTLGTEDTARYYC	TGGHRCYGLFGGCFGWFGDYYAMHL	WGPGVEVAVSS	IGHV1-14*01	92.36
IgG1-11B|11C|11D	EEKLVESGG.GLVQPGGSLRLSCVGS	GFDL....RRYG	VVWVRQAPGKGLESLAF	IGSVSYLGST	YYADSVK.GRFTISRDDSQNTVYLQLNSLRTEDTARYYC	ARVGCYCNGGSWYADRPVFMDL	WGPGVEVVVSS	IGHV1-11*01	94.56
IgG1-11E	EVKLVESGG.GLVQPGGSLRLSCVGS	GFDL....RRYG	VVWVRQAPGKGLESLAF	IGSVSYLGST	YYADSVK.GRFTISRDDSQNTVYLQLNSLRTEDTARYYC	ARVGCYCNGGSWYADRPVFMDL	WGPGVEVVVSS	IGHV1-11*01	94.22
IgG1-11F	EVKLVESGG.GLVQPGGSLRLSCVGS	GFDL....RRYG	VVWVRQAPGKGLESLAF	IGSVSYLGST	YYADSVK.GRFTISRDDSQNTVYLQLISLRTEDTARYYC	ARVGCYCNGGSWYADRPVFMDL	WGPGVEVVVSS	IGHV1-11*01	93.2
IgG1-12B|12D	EVKLVESGG.GLVQPGGSLRLSCVGS	GFDL....RRYG	VVWVRQAPGKGLESLAF	IGSVSYLGST	YYADSVK.GRFTISRDDSQNTVYLQLNSLRTEDTARYYC	ARVGCYCNGGSWYADRPVFMDL	WGPGVEVVVSS	IGHV1-11*01	94.56
**IgLC-κ-2G**	**AIVLTQTPLPLSVSPGEPASISCRSG**	**QSLLHT.DGANY**	**LNWYLQKPGRSPQLLIY**	**YA.......T**	**NRASGVP.DRFTGSG..SGTDFTLKISRVEAEDVGVFYC**	**FLSLQSPYG**	**FGAGTKLELK**	**IGKV2-10*02**	**93.54**
IgLC-λ-4D	SYELTQPSS.ESVALGKTAKITCSGD	LLD......KKY	TQWYQQKPGQAPLLVIY	KD.......S	ERPSGIP.ERFSGSS..SGKTATLSITGAQAEDEADYYC	QSADNIHNVI	FGGGTHLTVL	IGLV3-3*01	96.42
**IgLC-λ-5E**	**SYELTQPSS.ESVALGNTAKITCSGD**	**LLD......AKY**	**TNVEKRGGQAPPRLLIY**	**KD.......S**	**ERPSGIP.ERFSGSS..SGKTATLTITGAQAEDEAEYFC**	**QSADSLDSVP**	**FGGGTHLSVL**	**IGLV3-3*01**	**87.81**
**IgLC-λ-5F**	**SYELTQPSS.ESVALGNTAKITCSGD**	**LLD......AKY**	**TQWYQQKSGQAPLLLIY**	**KD.......T**	**ERPSGIP.ERFSGSS..SGKTATLTITGAQAEDEADYYC**	**QSADRIDNVP**	**FGGGTHLTVP**	**IGLV3-3*01**	**97.49**
**IgLC-λ-7A|7E**	**SYEVTQPSS.ESVALGNTAKITCSGD**	**LLD......KKY**	**TQWYQQKPGQAPLLLIY**	**KD.......S**	**ERPSGIP.ERFSGSN..SGKTATLTITGAQAEDEADYYC**	**QSADNIHNVI**	**FGGGTHLTVP**	**IGLV3-3*01**	**96.77**
**IgLC-λ-9D**	**SYELTQPAS.ESVALGNTAKITCSGD**	**LVD......KTG**	**TRWYQQKPGQAPLLLIY**	**ND.......S**	**ERPSGIP.ERFSGSN..SWRTATLTITGAQAEDEADYYC**	**RSPDISGHLV**	**FGGGTHLTVL**	**IGLV3-3*02**	**93.55**
IgLC-κ-11A	AIVLTQTPLSLSVSPGEPASISCRSS	LTLPT..NGAFG	KKEKSRNQASLHSSLSM	RL.......P	TGPLGSH.FPFSGSG..SGTDFTLKINRVEAEDAGVYYC	QQFKEFPRG	FGAGTKLELK	IGKV2-13*02	70.79
IgLC-κ-11B	AIVLTQTPLSLSVSPGEPASISCRSS	QSRCRR.MEHLR	KKRGEQKPGQSPQLLIY	EA.......T	NRASGVP.YPLSGSG..SGTDFTLKINRVEAEDAGVYYC	QQFKEFPRG	FGAGTKLELK	IGKV2-13*02	68.42
IgLC-κ-11C	AIVLTQTPLSLSVSPGEPASISCRSS	QSDAA..DEWNI	EKGKQQKPGQSPQLLIY	EA.......T	NRASGVP.DRFSGSG..SGTDFTLKINRVEAEDAGVYYC	QQFKEFPRG	FGAGTKLELK	IGKV2-13*02	69.1
IgLC-κ-11D	AIQLTQSPASLAASLGDTVSITWRAS	QSDAAD.EWSLW	DKGWKKKPGQSPQLLIY	EA.......T	NRASGVP.FPFSGSG..SGTDFTLKINRVEADDAGVYYC	RQFKEFPRG	FGAGTKLELK	IGKV2-13*02	67.01
IgLC-κ-11E	AIVLTQTPLSLSVSPGEPASISCRSS	ASDAA..DEPYR	PKKKRRNRPVSTLLIYE	AT.......N	RASGVPS.LRKKGSG..SGFDFTLKINRVEAEDAGVYYC	QQFKEFPRG	FGAGTKLELK	IGKV2-13*02	67.01
IgLC-κ-11F	AIVLTQTPLSLSVSPGEPASISCRSS	AHLTL..PTNGH	WQKRRRNRPVSTAPDLY	GY.......Q	QGLWGPI.PRFSGSG..SGTDFTLKINRVEVEDAGVYYC	QQFKEFPRG	FGAGTKLELK	IGKV2-13*02	68.04
IgLC-κ-12C	AIVLTQTPLSLSVSPGEPASISCRST	SDAADE.WRFDN	KKKGAETRPVSTAPDLY	GY.......Q	QGLWGPS.PPFSGSG..SGSDFTLKINRVEAEDAGVYYC	QQFKEFPRG	FGAGTKLELK	IGKV2-13*02	68.04
IgLC-κ-12D	AIVLTQTPLSLSVSPGEPASISCRSS	QSRCR..RMEAI	CHEYQQKPGQSPQLLIY	EA.......T	NRASGVP.DRFSGSG..SGTDFTLKINRVEAEDAGVYYC	QQFKEFPRG	FGAGTKLELK	IGKV2-13*02	71.93

Table S4: Protein display of in-frame IGV for sequenced single cell pair IGH and IGLC genes using IMGT unique numbering. Sequences from single sorted B cells were compared to standardized representations of the amino acid sequences of the V-DOMAINs, including framework regions (FR1-IMGT, FR2-IMGT, FR3-IMGT, FR4-IMGT) and complementarity determining regions (CDR1-IMGT, CDR2-IMGT, CDR3-IMGT), enabling comparison to, and calculation of distance from, nearest germline sequences. Sequences in bold were successfully expressed.

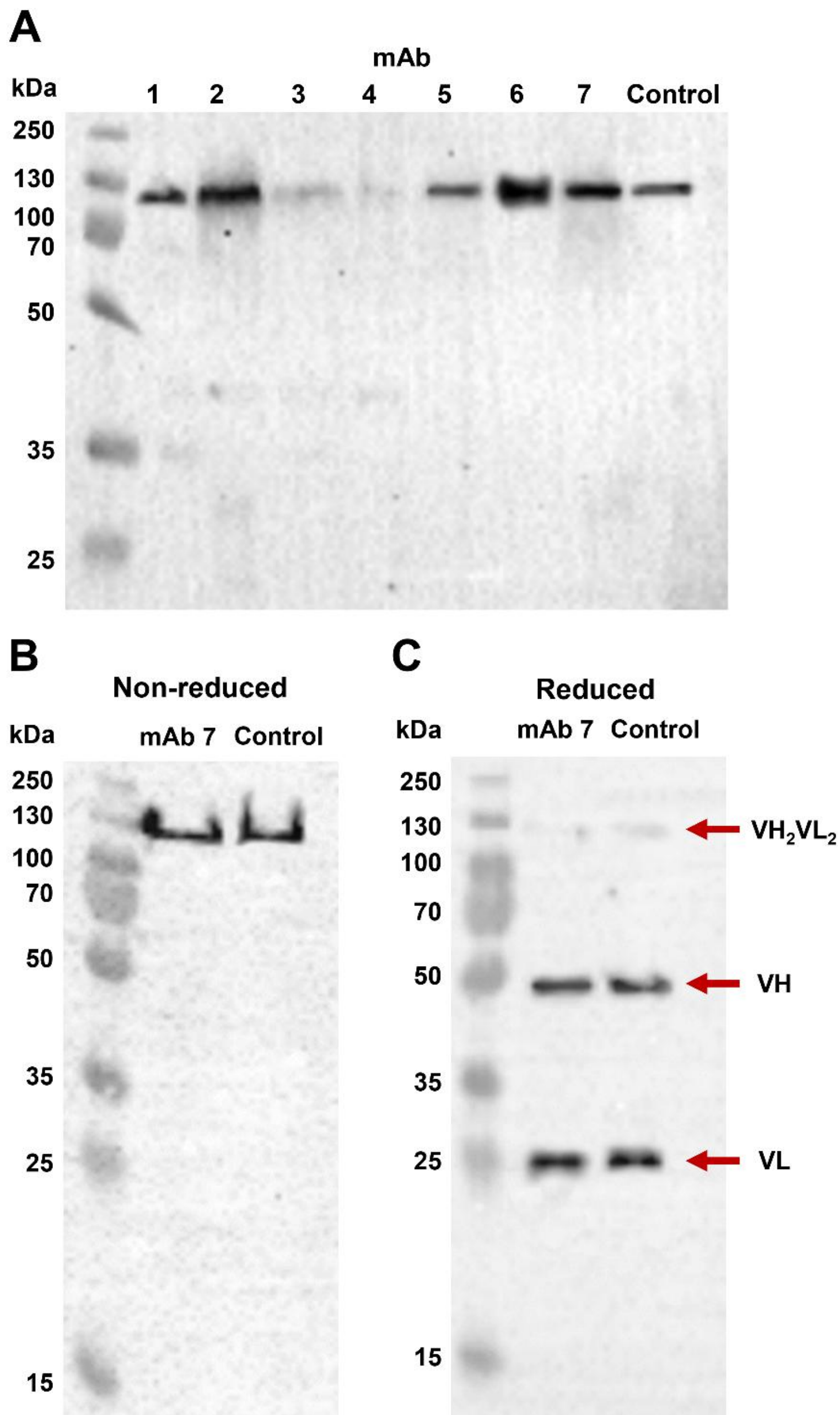

Figure S7: Recombinant expression of porcine mAbs. Expression of mAbs from transfected Expi293F cells was assessed by western blot (A). Representative gels comparing mAb 7 and a Nipah virus glycoprotein-specific control mAb as shown under reducing and non-reducing conditions (B, C). Some larger bands could still be detected, comparable to the non-reduced samples, likely suggesting that there had been incomplete reduction of the mAbs.

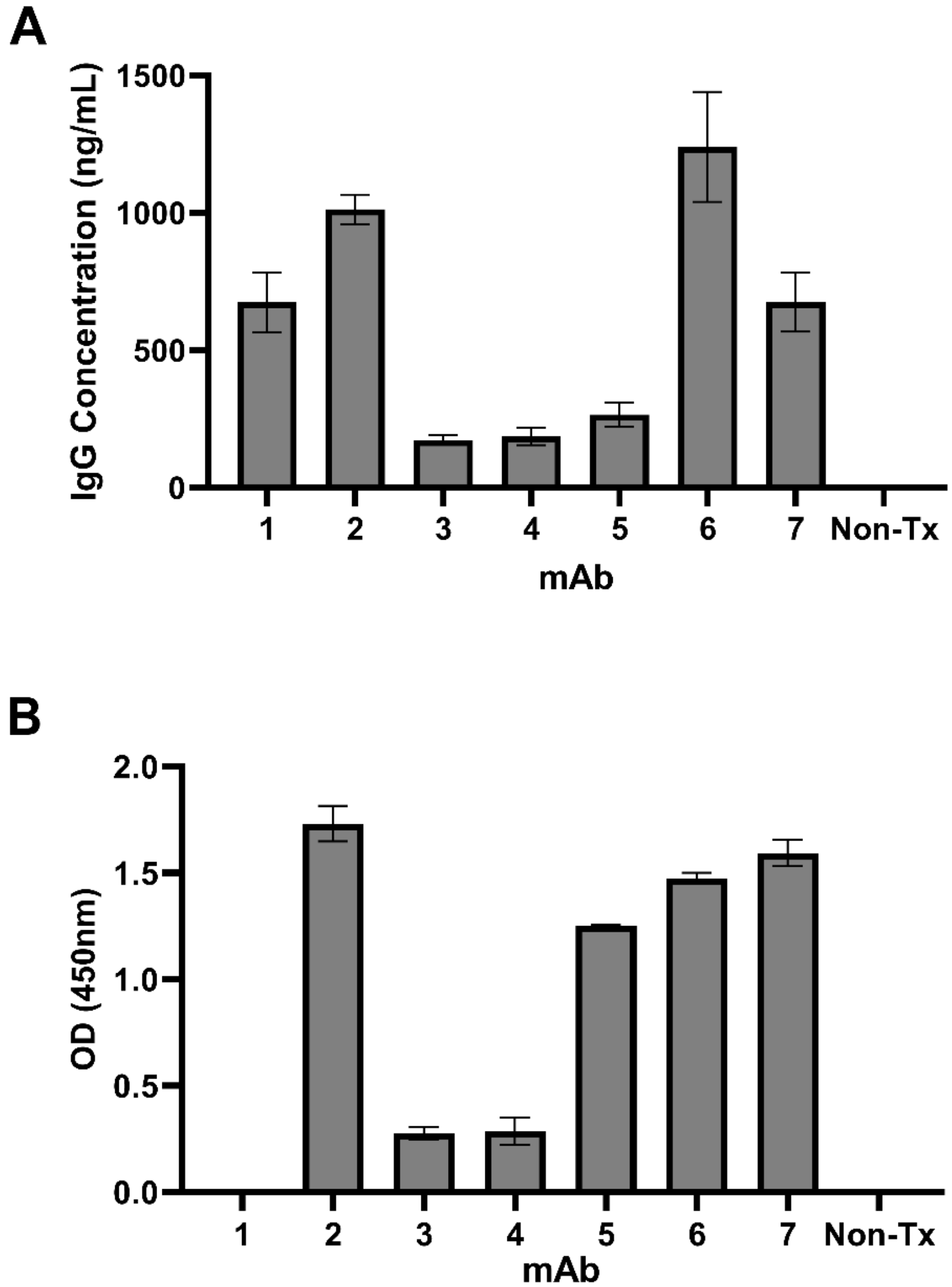

Figure S8: Assessment of concentration and specificity of porcine mAbs in culture supernatants from transfected Expi293F cells. Following transfection, supernatants were screened by ELISA using an anti-pig IgG (H+L) secondary antibody, and OD values interpolated against a porcine IgG standard curve to determine concentration (A). Supernatants were also evaluated by ELISA for binding to PRRSV-1 sGP3 (B).

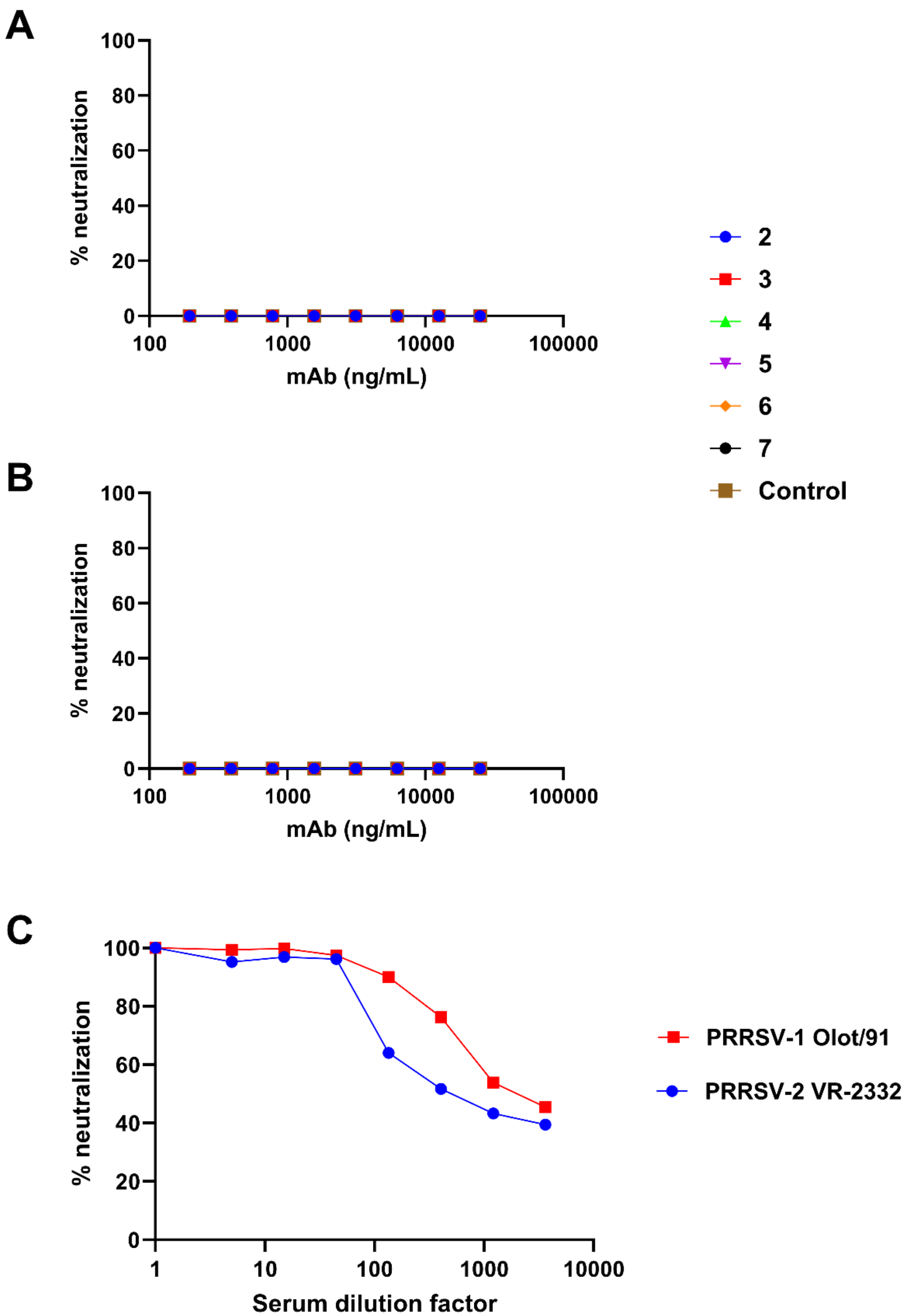

Figure S9: Neutralization capacity of PRRSV sGP3-specific mAbs was assessed by VNT. Each mAb was incubated with either PRRSV-1 Olot/91 (A) or PRRSV-2 VR-2332 (B), before being added to MARC-145 cells. Serum from hyperimmunized pig 64 was included in both experiments as a positive control (C). Percentage neutralization was calculated based on detection of PRRSV nucleocapsid by immunofluorescence staining.

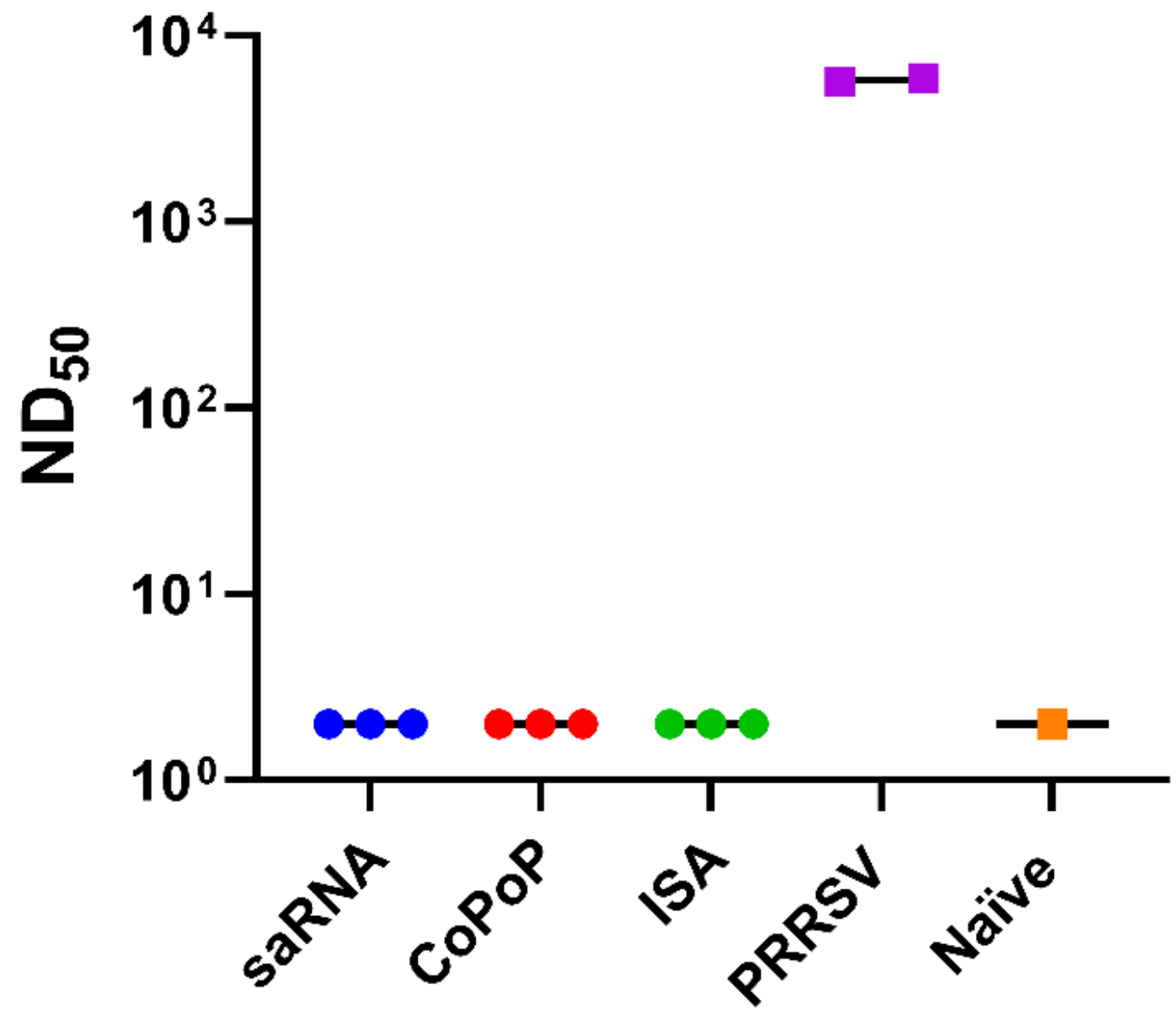

Figure S10: Neutralization capacity of PRRSV-1 GP3-immunized pigs by primary cell VNT. The neutralization of PRRSV-1 Olot/91 infection in porcine alveolar macrophages was determined from 56-days post-vaccination (dpv) serum samples from pigs immunized with sGP3 displayed on adjuvanted liposomes (CoPoP), saRNA vectored membrane displayed GP3 (saRNA), or sGP3 formulated with Montanide ISA 201 VG adjuvant (ISA), and compared to a PRRSV immune pig (PRRSV) and a naïve animal (naïve). Mean data from three biological replicates ± SD are shown.

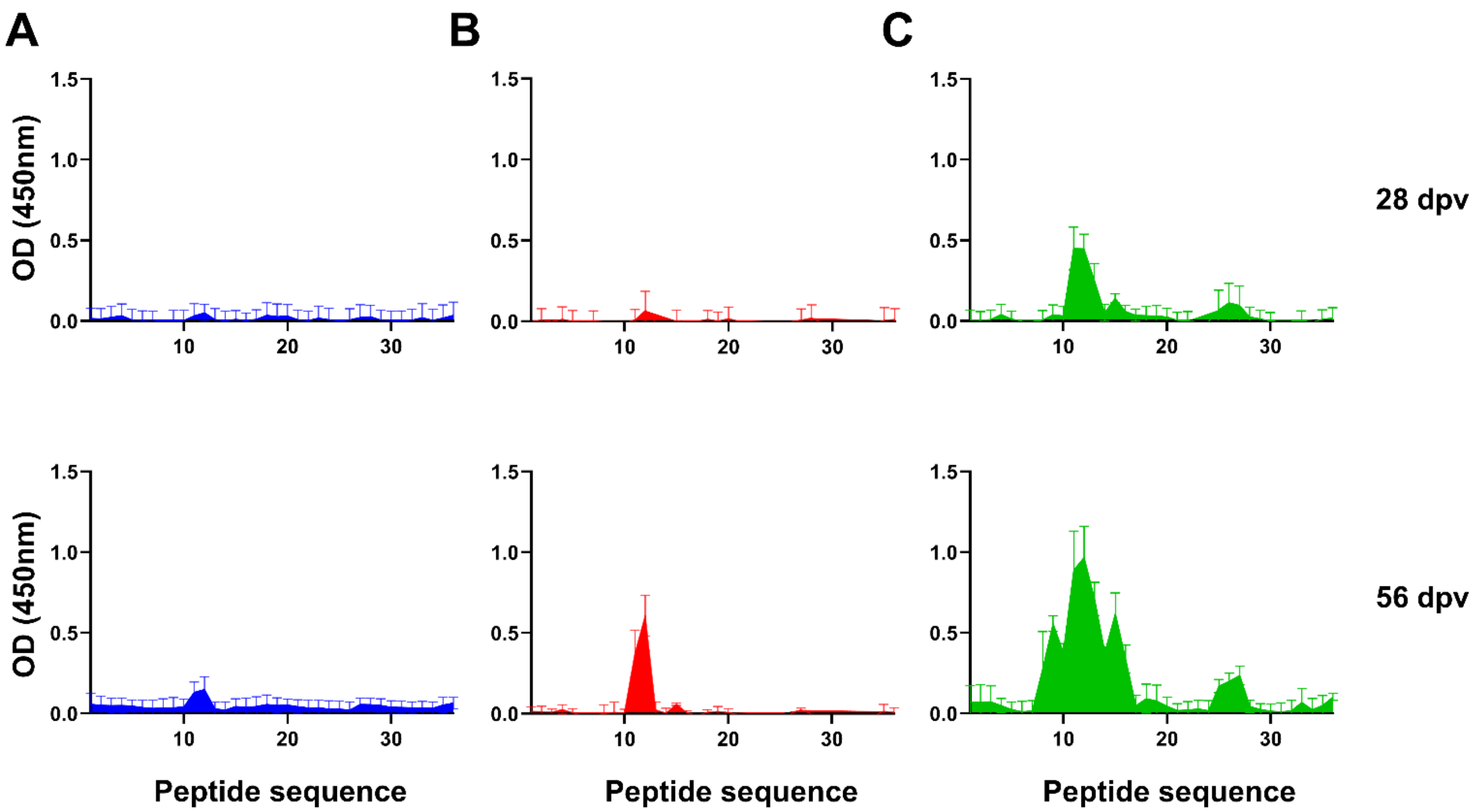

Figure S11: Recognition of linear epitopes by sera from PRRSV-1 GP3-immunized pigs. Sera from 28- and 56-days post-vaccination (dpv) from pigs immunized with sGP3 displayed on adjuvanted liposomes (CoPoP) (A), saRNA vectored membrane displayed GP3 (saRNA) (B), or sGP3 formulated with Montanide ISA 201 VG adjuvant (ISA) (C), were compared. Mean data of two technical replicates from three biological replicates ± SD are shown.
